# Structural Colors of Cyclic Diblock Copolymers Enabled by Topology-Driven Structural Ordering and Tuned by Blending with Constituent Cyclic Polymers

**DOI:** 10.1021/jacs.5c21376

**Published:** 2026-02-27

**Authors:** Jiyun Nam, Eugene Y.-X. Chen

**Affiliations:** Department of Chemistry, 3447 Colorado State University; Fort Collins, Colorado 80523, United States

## Abstract

Controlling structural colors of high-molecular-weight (HMW) block copolymers is challenging due to their pronounced chain entanglements, often limiting accessible polymeric architectures required for self-assembly into photonic crystals. Here, we demonstrate tunable structural colors in photonic films induced by the self-assembly of simple cyclic diblock copolymers (CDCs). Thanks to the unique compounded sequence and spatiotemporal topology controls of Lewis pair polymerization, one-pot copolymerization of (meth)­acrylic monomer mixtures furnishes the precision synthesis of well-defined CDCs with a HMW (862.4 kDa) at scale (20 g). The resulting CDC spontaneously self-assembles into a well-ordered lamellar structure, producing a vivid blue structural color. In situ small-angle X-ray scattering measurements reveal that the self-assembly of the HMW CDC occurs at a much faster rate than that of its linear counterpart, leading to long-range ordering with improved lamellar domain purity and avoiding a kinetically trapped morphology. Blending the CDC with the linear counterpart disrupts this coloration, coinciding with a loss of lamellar structural ordering. Owing to the cyclic topology-driven structural ordering, blending the CDC with cyclic polymer additives swells the lamellae up to 123% of the initial spacing, allowing for controlling the reflected wavelength spanning from 467 to 608 nm and accordingly producing blue to orange colors.

## Graphical abstract

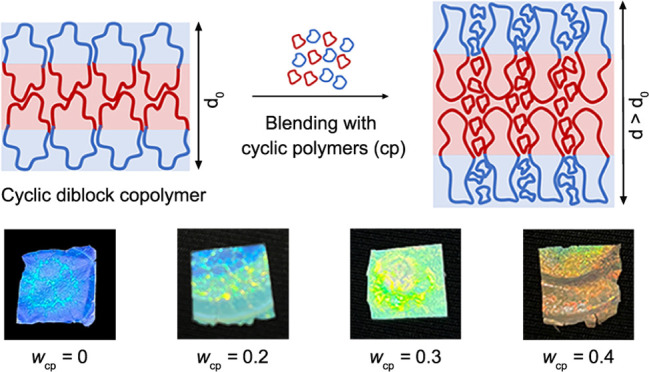

## Introduction

Structural colors in nature, appearing in butterfly wings, peacock feathers, and chameleon skins, offer a sustainable and durable way of coloration.^1–3^ Their colors arise from light interaction of periodic refractive index modulation, such as reflection and diffraction through arranged or ordered nanostructures, which is fundamentally different than synthetic dyes that absorb specific wavelengths of light by their chromophores.^4,5^ To mimic bioinspired approaches, photonic crystals (PCs) are composed of periodic arrays containing more than two domain layers with different refractive indices. Bragg diffraction of incident light through a multilayered structure induces a forbidden frequency zone, which results in a strong reflection of light with a specific wavelength, appearing in vivid colors.^6^ Self-assembly of block copolymers affords bottom-up approaches for organic PCs when their structural periodicities with different refractive indices are comparable to the wavelength of light.^7,8^ However, achieving structural coloration spanning the visible spectrum requires over 100 nm of domain spacing, which has been challenging to achieve by diblock copolymer assembly. The chain entanglement of the high-molecular-weight (HMW) polymer increases the kinetic barrier of the assembly process. The hindered diffusion and rearrangement of the chains result in phase separation into a kinetically trapped morphology, limiting access to domain sizes large enough to reflect visible light.^9,10^

Effective strategies developed to suppress the inherent chain entanglement resulted from harnessing the structure–property relationship of specifically designed polymer architectures. One such example is based on bottlebrush polymers (BBPs), where the steric hindrance of densely grafted side chains extends the backbone.^11–13^ The resulting worm-like conformation largely suppresses or completely avoids the chain entanglement, thus promoting rapid self-assembly, and expands the accessible lamellar domain spacing up to several hundred nanometers. Therefore, the reflected wavelength of BBP-based PCs can be broadly tuned across UV–vis–IR by controlling the lamellar spacing, depending on their degree of polymerization (DP).^14–17^ Another potential strategy is to produce a ring architecture that is absent of free chain ends in the case of cyclic polymers (CPs). Intermolecular sliding of double-folded loops in CP melts enables faster stress relaxation than in linear polymers.^18–20^ In addition, the compact cyclic topology reduces CP’s conformational freedom, which enhances intermolecular interaction through compact packing, thus promoting the formation of a highly ordered nanostructure.^21^ Because of their compactness, however, CPs generally produce nanostructures with reduced domain spacing, theoretically predicted to be 30–37% more compact than that of linear analogues.^21–25^ Therefore, achieving well-defined nanostructures exhibiting over 100 nm domain spacing has been hindered via self-assembly of cyclic diblock copolymer (CDC) systems due to the required much higher MW of CPs than their linear analogues.

The challenge in the synthesis of well-defined CPs with HMW (>100 kDa) lies in the ring-closure step through a direct coupling reaction between two opposite, reactive chain ends.^26^ By introducing a coupling functional group (e.g., alkyne) and a complementary group (e.g., azide) at the opposite chain termini through postpolymerization modification, the heterodifunctional polymer undergoes ex situ cyclization via the click reaction.^27,28^ To enhance the selectivity for intramolecular cyclization over the competing intermolecular coupling, this method requires a highly diluted polymer solution, which severely reduces the effective concentration of reactive end groups.^29,30^ Furthermore, localizing the two reactive ends of a long polymeric chain under dilute conditions significantly increases the entropic penalty. Overall, the cyclization of the HMW polymer is thermodynamically disfavored via ex situ coupling reaction. Another route involves in situ cyclization via coordinative^31–33^ and zwitterionic ring-opening polymerization.^34–36^ In conventional anionic polymerization, the lowering propagation rate (*k*_p_) throughout the chain growth step competes with the rate of ring-closing chain transfer (*k*_tr_), CPs with less-controlled MW and broad dispersity (*D̵*) were produced by the statistical ring closure.^37–39^ In contrast, Lewis pair polymerization (LPP) provides CPs with controlled MW and low *D̵* values, thanks to the zeroth-order dependence of *k*_p_ on monomer concentration and spatiotemporal control on cyclization.^40–42^ The unique feature of *k*_p_ in LPP, which is proportional to [LA]_0_[LB]_0_ (LA = Lewis acid, LB = Lewis base), enables *k*_p_ ≫ *k*_tr_ such that the chain transfer (i.e., ring closure) can occur only after complete monomer consumption.^37,40^

Herein, we explored the self-assembly of simple acrylic HMW CDCs toward PCs, along with comparative studies on linear diblock copolymer counterparts, both of which are uniquely accessible by the LPP with compounded sequence and spatiotemporal topology controls (Figure 1A).^41,43–46^ The resulting CDC phase separated into a lamellar structure over 100 nm in long-range order, the bulk film of which reflected a vivid blue color. The linear counterpart, however, shows a less-ordered phase-separated structure, resulting in the diffuse reflectance spectra being opaque in the visible. In addition, blending medium-MW CP additives corresponding to each block into the CDC allows for swelling of the lamellar domain between 172 and 224 nm while keeping the lateral order of the nanostructure with high domain purity, conveniently and effectively tuning CDC-based structural colors across the visible spectrum range, from blue to green to orange colors (Figure 1B).

**Figure 1:**
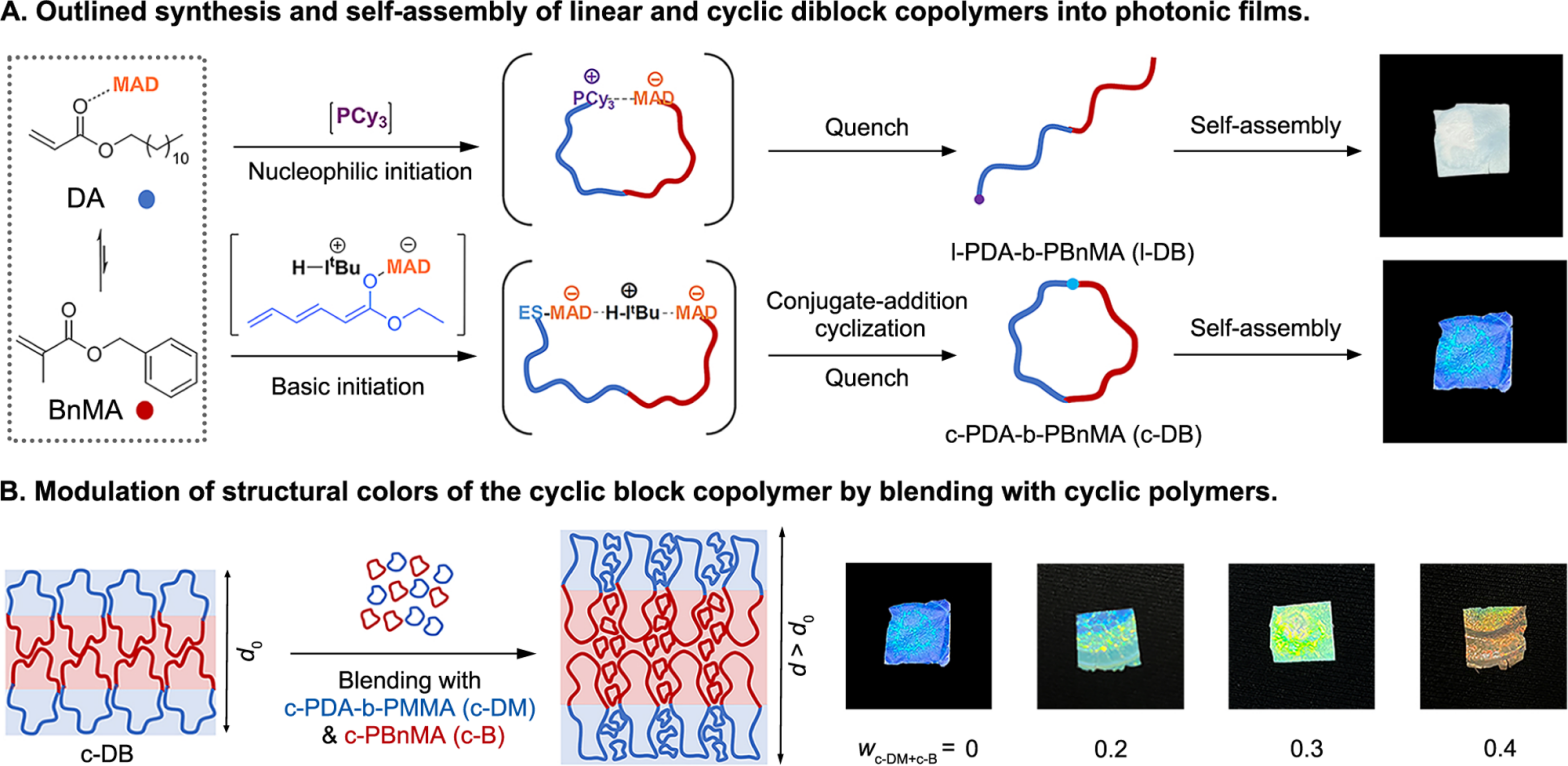
(A) Compounded thermodynamic and kinetic comonomer sequence and spatiotemporal topology controls in LPP for the synthesis of the CDC and its linear counterpart and their comparative self-assembly behavior toward photonic films. (B) Tuning of structural colors through swelling of lamellar domain spacing by adding CP additives (c-DM and c-B) at various weight fractions (*w*_c‑DM+c‑B_ = 0–0.4).

## Results and Discussion

### Design, One-Pot Synthesis, and Characterization of All-Acrylate Photonic CDCs

Poly­(dodecyl acrylate) (PDA) and poly­(benzyl methacrylate) (PBnMA) were chosen as the two blocks in an all-acrylate photonic CDC due to their incompatibility, which would promote spontaneous phase separation for 1-D periodic morphology as Bragg stacks, as predicted based on their difference in Hansen solubility parameters. For PBnMA, the solubility parameter is reported to be 20.8 MPa^1/2^.^47^ For PDA, we estimated the solubility parameter to be smaller than 17.3 MPa^1/2^ of poly­(butyl acrylate), considering PDA’s even longer nonpolar alkyl chain.^48^ In addition, we expected PDA-*b*-PBnMA photonic films to exhibit high reflectance arising from relatively high refractive index (*n*) contrasts between two blocks: PDA served as the low *n* block (*n*_PDA_ ∼ *n*_PDMA_ = 1.474 (PDMA = poly­(dodecyl methacrylate)),^49^ which has a lower *n* than poly­(methyl methacrylate) (PMMA) (*n*_PMMA_ = 1.489),^49^ whereas PBnMA formed the high *n* block (*n*_PBnMA_ = 1.568).^50^

By employing the compounded sequence and spatiotemporal topology-controlled LPP methodology,^43–46^ we first assessed the synthesis of the CDC and its linear counterpart from one-pot LPP of the comonomer mixture. To obtain compositionally identical but topologically distinctive linear and cyclic block copolymers, different initiating species were employed: LB tricyclohexylphosphine (PCy_3_) and LA methyl aluminum di­(2,6-di*tert*-butyl-4-methylphenoxy) (MAD) for the linear diblock polymer synthesis and an ion pair derived in situ from a stoichiometric ratio of ethyl sorbate (ES)/LA (MAD)/LB {1,3-di*tert*-butylimidazol-2-ylidene (I^*t*^Bu)} for the CDC synthesis.^45^ For clarity, we denote the PDA-*b*-PBnMA diblock copolymer as l-DB_*x*_ and c-DB_*x*_ for linear and cyclic diblock copolymers, respectively, where x represents DP. Polymerization reaction mixtures containing DA, BnMA, and MAD were prepared such that a symmetric weight fraction of blocks was kept (Table S1).

Probing the dynamic mixture of DA, BnMA, and MAD by variable-temperature NMR revealed no observable coordination between BnMA and MAD even at −15 °C, implying a heavily biased equilibrium constant (*K*_eq_ > 1000) for MAD coordination to DA over BnMA (Figure S1).^51^ Additionally, a significantly (∼400 times) faster propagation rate (*k*_p, obs_) was obtained for DA polymerization (*k*_p, obs_ = 1.28 × 10^5^ [M]·s^–1^·[MAD]_*t*_^–1^·[LB]_0_^–1^) than that for BnMA polymerization (*k*_p, obs_ = 3.2 × 10^2^ [M]·s^–1^·[MAD]_*t*_^–1^·[LB]_0_^–1^) (Figure S2). These results suggest constructively compounded thermodynamic (*K*_eq_) and kinetic (*k*_p_) sequence control in the LPP of the DA and BnMA mixture for the formation of a well-defined block copolymer. Indeed, by adding the initiating species to the above mixture, the combined much superior MAD affinity and higher rate of polymerization for DA over BnMA rendered exclusive addition of DA to construct the PDA block first, followed by polymerization of BnMA only after complete consumption (<5 s) of DA, as depicted in Figures 2A and S3, producing c-DB_2800_ and l-DB_2800_, the topology of which depends on the initiating species (vide supra).

**Figure 2:**
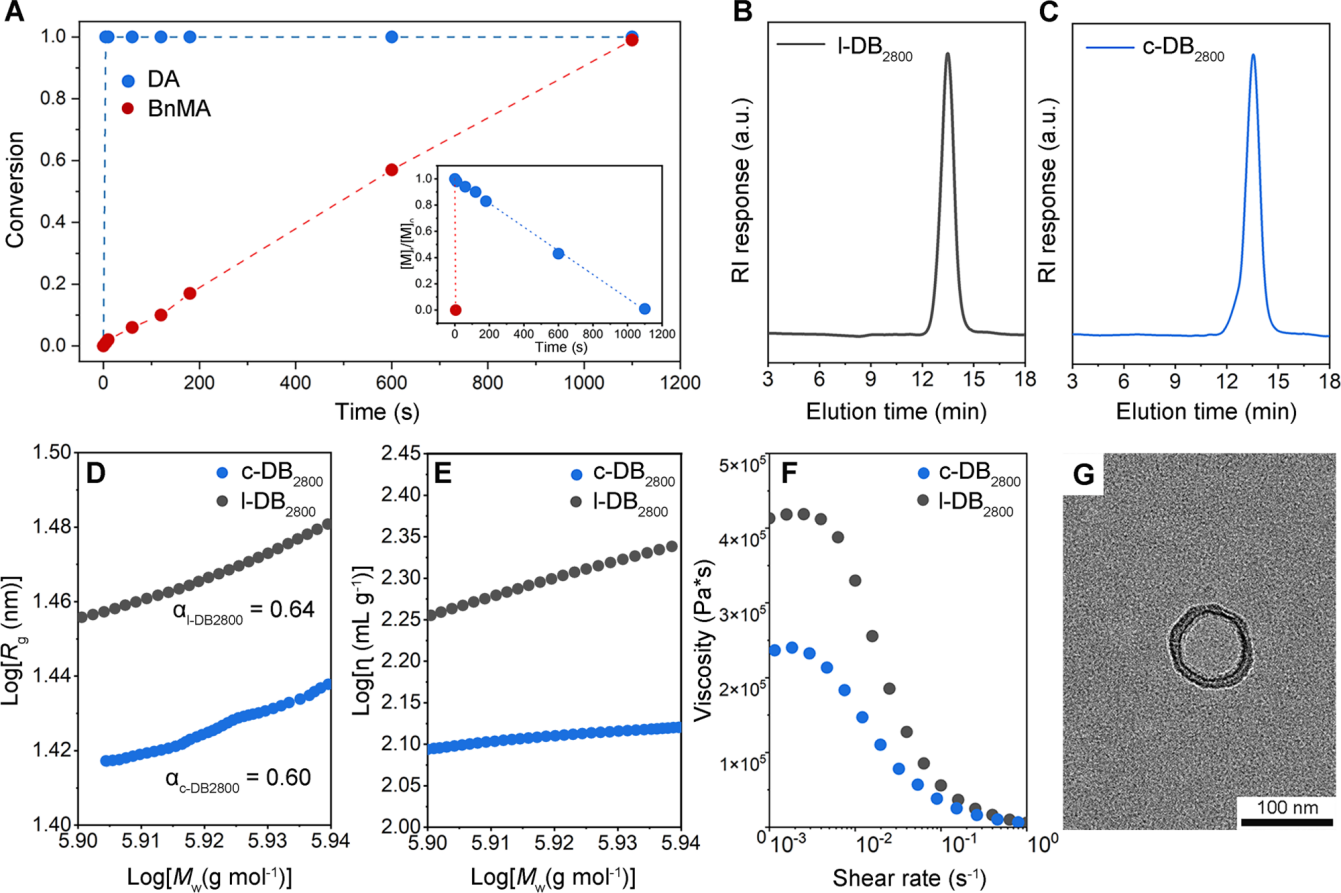
(A) DA (blue dots) and BnMA (red dots) conversion data during the LPP for the synthesis of c-DB_2800_. Inset: plot of zero-order kinetics in toluene at room temperature (RT): [DA]_0_ = 0.22 M; [BnMA]_0_ = 0.38 M; [MAD]_0_ = 0.006 M; [ES-IP]_0_ = 0.00021 M. (B) SEC trace of l-DB_2800_. (C) SEC trace of c-DB_2800_. (D) Plots of *R*_g_ as a function of *M*_w_ for c-DB_2800_ and l-DB_2800_. (E) Mark–Houwink–Sakurada plots for c-DB_2800_ and l-DB_2800_. (F) Zero shear viscosity of c-DB_2800_ and l-DB_2800_ at 195 °C. (G) Representative TEM image of c-DB_2800_, confirming its cyclic topology. The specimen was prepared with a polymer concentration of 0.1 mg mL^–1^ in toluene.

While the LPP reaction was complete within 20 min, the reaction mixture was left for longer times to complete the much slower cyclization via conjugate addition.^41^ The disappearance of the proton signal of the terminal alkenyl moiety was observed in the ^1^H NMR spectra of the low-DP BnMA polymerization mixture (BnMA/MAD/ES-IP = 40/2/1, 0.5 M in toluene) in Figure S4. This data indicated that the conjugate addition of the BnMA enolate terminus to the vinyl end group of the ES-derived initiating species had occurred in completion. Multi (18)-angle light scattering-size-exclusion chromatography (MALS-SEC) analysis showed high weight-average molar mass (*M*_w_) polymers of c-DB_2800_ (*M*_w_ = 862.4 kDa) and l-DB_2800_ (*M*_w_ = 879.3 kDa) with a narrow dispersity (*D̵* = 1.02–1.06) were produced at a 20 g scale in a one-pot procedure (Table S2, Figures S5 and S6). The high degree of block resolution of l-DB_2800_ and c-DB_2800_ was confirmed by two distinctive glass-transition temperature (*T*_g_) domains and the well-resolved ^13^C NMR signals of each block characteristic of the peak corresponding to homopolymers (Figures S7 and S8). Integration ratios of proton signals for DA and BnMA repeat units in ^1^H NMR spectra confirmed the identical block composition of l-DB_2800_ and c-DB_2800_ (Figures S9 and S10).

We further characterized the topological differences between l-DB_2800_ and c-DB_2800_ in dilute CHCl_3_ solution with MALS-SEC. The obtained refractive index increment (d*n*/d*c*) showed a lower d*n*/d*c* value for c-DB_2800_ (d*n*/d*c* = 0.0719 mL/g) than that of l-DB_2800_ (d*n*/d*c* = 0.0857 mL/g) (Figures S5 and S6), consistent with the previous finding.^45^ With these values, the MW of linear and cyclic PDA-*b*-PBnMA showed a marginal difference in *M*_w_, indicating similar compositional characteristics (Table S2). SEC traces of c-DB_2800_ and l-DB_2800_ showed a shift of the peak maximum to the lower molar mass region with a ratio of *M*_p,c‑DB_/*M*_p,l‑DB_ = 0.96, as shown in Figure 2B,C. Although the conformational scaling relationship (*R*_g_–*M*_w_^α^) suggested a slightly smaller α for c-DB_2800_ than for the linear analogue, MALS-SEC determined the considerably smaller hydrodynamic radius of gyration (*R*_g_) for c-DB_2800_ than for l-DB_2800_ with *g* = *R*_g_,_c‑DB_^2^/*R*_g_,_l‑DB_^2^ ∼0.65 (Figure 2D and Table S2), consistent with the reported *g* factor for cyclic versus linear polymers.^34,52,53^ Mark–Houwink–Sakurada (MHS) plots (Figure 2E) further revealed intrinsic viscosities of c-DB_2800_ much lower than those of the linear analogue, [η]_c‑DB_/[η]_l‑DB_ = ca. 0.65. The reduction in both *R*_g_ and [η] clearly indicated that c-DB_2800_ adopted a conformation that was more compact than that of l-DB_2800._ ^54^ A much lower zero-shear viscosity (η_0_) of polymer melts was demonstrated for c-DB_2800_, compared to l-DB_2800_, η_0_, _c‑DB_/η_0,l‑DB_ = ca. 0.57 (Figure 2F), indicating a lower degree of chain entanglement for the CDC due to the absence of free chain ends. Furthermore, the cyclic topology of c-DB_2800_ with a diameter of ∼82 nm was confirmed by transmission electron microscopy (TEM) imaging (Figures 2G and S11 for the low-magnification TEM images). We attributed the skeletal ring-like morphology observed in TEM to a stiff and locally extended backbone conformation in the c-DB_2800._ The clearly defined backbone correlation peak of c-DB_2800_ in wide-angle X-ray scattering implied that a relatively long alkyl chain length of PDA side groups (contour length, *L*_c_ = 21.5 Å) compared with the polyacrylate backbone unit (*L*_c_ = 3.1 Å) imposed the steric hindrance that promoted local backbone extension (Figure S12).^55,56^

Overall, the above comprehensive characterizations confirmed the formation of the target CDC and its linear counterparts by the LPP method.

### Self-Assembly of PDA-*b*-PBnMA CDC into Photonic Films

To test the hypothesis that the suppressed chain entanglement in CDC would lower the kinetic barrier of the self-assembling process and thus lead to notably different morphological features responsible for discrete photonic properties when compared to its linear counterpart, we investigated the kinetics of self-assembly of as-prepared, solution-casted linear and cyclic PDA-*b*-PBnMA bulk films by in situ time-resolved small-angle X-ray scattering (SAXS).^59^ As shown in Figure 3A, c-DB_2800_ with the equal weight fraction of PDA and PBnMA blocks (*w*_PDA_ *= w*_PBnMA_) exhibited a primary peak at q* = 0.03481 nm^–1^, followed by higher-order scattering functions at *q*/q* = 3 and 5 in the as-prepared film at 25 °C. Upon annealing at 80 °C for 20 min, an increase in peak intensity was observed, and a higher-order peak of 7q* appeared. Further increment of peak intensity was induced by annealing for 130 min. In comparison, ex situ thermal annealing at 120 °C for 24 h resulted in the development of an intense primary scattering peak at 0.03651 nm^–1^, followed by improved development of highly ordered scattering functions at *q*/q* = 3, 5, 7, and 9 (Figure 3C). This observation indicated that a highly ordered symmetric lamellar morphology was formed along with long-range ordering and high phase purity in c-DB_2800_. In comparison, a disordered morphology for l-DB_2800_ was discerned from the broad peaks at the initial stage, as shown in Figure 3B. In addition, l-DB_2800_ only provided the ill-defined phase-separated structure with a broad principal peak at 0.0280 nm^–1^ even after ex situ annealing for 24 h at 120 °C.

**Figure 3:**
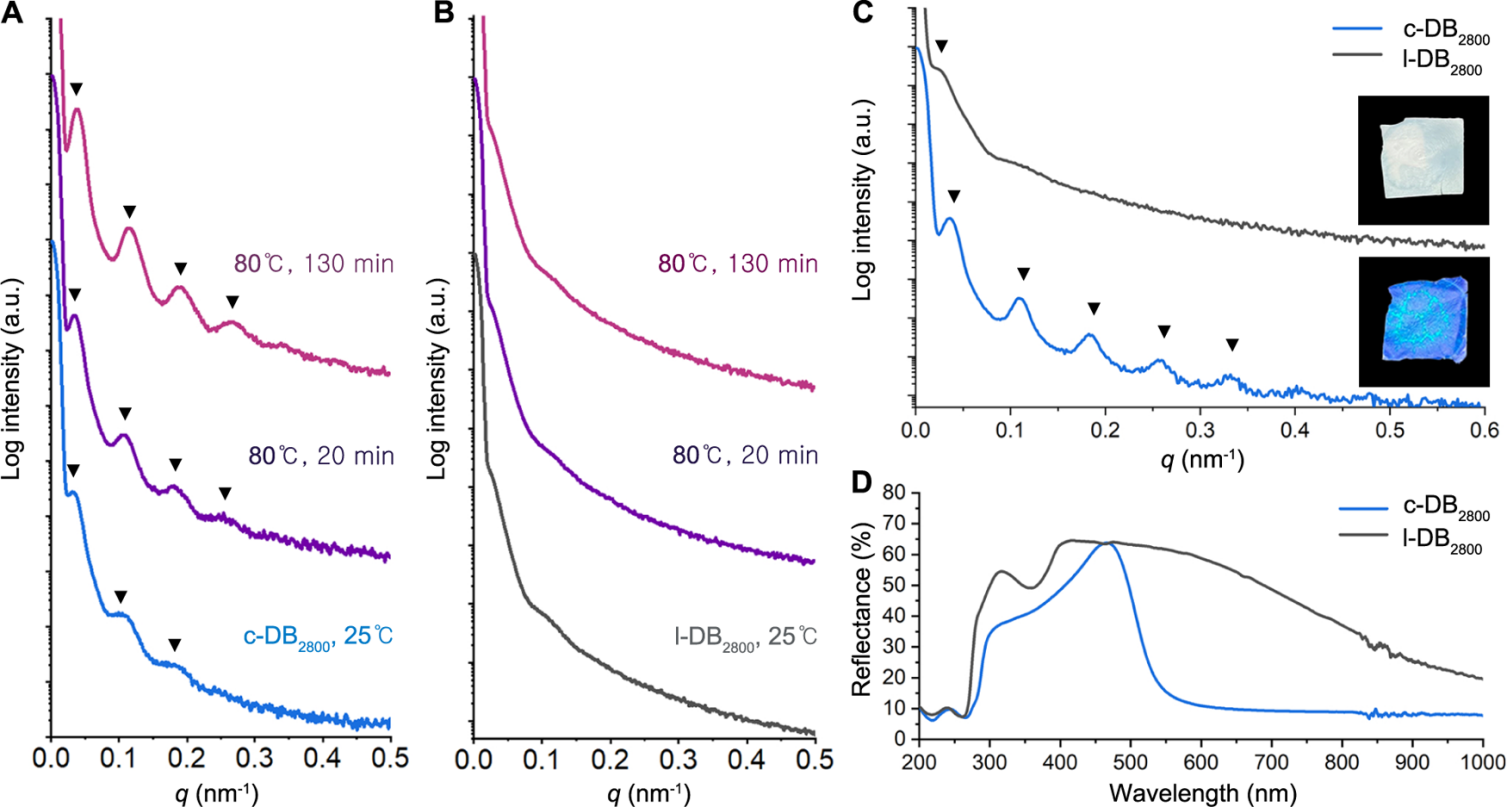
In situ time-resolved SAXS patterns for c-DB_2800_ (A) and l-DB_2800_ (B). The bulk samples were prepared by solvent casting with THF and evaporation at RT. (C) SAXS profiles of c-DB_2800_ and l-DB_2800_. The bulk films were prepared by THF solvent casting and ex situ thermally annealed at 120 °C for 24 h under a vacuum. Insets show photographs of the corresponding bulk films of c-DB_2800_ (bottom) and l-DB_2800_ (top). The SAXS patterns were vertically shifted for clarity. (D) Plots of reflectance as a function of wavelength for c-DB_2800_ and l-DB_2800_.

The observed much faster assembly behavior of c-DB_2800_ compared to l-DB_2800_ implied that a reduced number of entanglements between cyclic chains lowered the kinetic barrier of rearrangement, producing a well-ordered lamellar structure over 100 nm domain spacing even at RT.^18–21^ This result indicated that the compact cyclic topology suppressed interdigitation between chains within domains, thereby reducing the interdomain entanglement and lowering the kinetic barrier for chain rearrangement during self-assembly. In addition, denser side-chain packing in confined cyclic topology promoted an additional enthalpic gain through more favorable side-group contacts, which together promoted the formation of highly uniform lamellar structures.^21,57–59^ In contrast, l-DB_1400_, which served as a linear analogue possessing an effective physical length comparable to c-DB_2800_ (Table S2 and Figure S14), exhibited the lamellar structure with a broad and less intense principal peak at 0.0483 nm^–1^, followed only by 3q* reflection after annealing at 120 °C for 24 h. Collectively, this result corroborated that the less entangled and compact cyclic topology primarily contributed to obtaining a well-ordered lamellar structure. Domain spacings (*d*) of lamellar structures were determined to be 172 nm for c-DB_2800_ and 224 nm for l-DB_2800_ based on the relationship of *d* = 2π/q* (Figures 3C and S15). While c-DB_2800_ exhibited 23% reduced *d* spacing due to its compact structure compared to the linear analogue, the well-defined nanostructure of the bulk c-DB_2800_ film allowed for reflection of vivid blue color (Figure 3C). Compared to c-DB_2800_, l-BDB_2800_ exhibited a 20% *d* reduction from l-DB_2800_, which was attributed to looping and the bridging of two opposite B end blocks (Figures S14–S16).^60,61^ A 42% decrease in *d* for l-DB_1400_ was observed due to half of the *M*_w_ scale compared to that of l-DB_2800_ (Figures S13−S16). All of the linear counterparts formed less-ordered morphologies with weak or absent high-order scattering functions, and their bulk films appeared opaque and white. The distinctively different photonic properties of the linear and cyclic PDA-*b*-PBnMA bulk films with similar thickness (0.21 ± 0.01 mm) were determined by UV–vis reflection spectra shown in Figure 3D. In the case of l-DB_2800_, extremely broad reflectance was observed, ranging from 260 to 1000 nm, with a reflectance peak of maximum intensity (λ_max_) of 420 nm. On the other hand, c-DB_2800_ bulk film showed primary reflectance at λ_max_ = 467 nm, with a significantly narrower peak width, resulting in a vibrant blue structural color. While a shift of the λ_max_ peaks was observed depending on the DP of c-DB (1400 and 2800), corresponding to increased *d* of lamellar, c-DB_2800_ was selected as the representative entry for subsequent studies due to its well-defined photonic response within the visible spectrum (see characterization details for Table S2 and Figures S17–S19).

### Tuning Structural Colors of Photonic CDC via Blending with CP

The reflected wavelength to microstructural parameters is governed by the equation of (1), where λ represents the peak reflected wavelength, *d*_1_ and *d*_2_ represent the thickness of the PDA and PBnMA domain layers, respectively, and *n*_1_ and *n*_2_ represent the refractive indices of each layer.^14^

1 $$\lambda =2\left(n_{1}d_{1}+n_{2}d_{2}\right)$$

Although the identical composition of c-DB_2800_ and l-DB_2800_ gives the same value of bulk refractive index of each layer and refractive index contrast (Δ*n*), which is *n*_1_ for PDA and *n*_2_ for PBnMA, the difference in structural ordering in the phase-separated structure resulted from the distinctive topology of building blocks (cyclic vs linear) leads to drastically different photonic behavior. We postulated that a well-ordered lamellar structure with a pure domain and high lateral ordering influences effective refractive index (*n*_eff_) and effective optical thickness (*d*_eff_), where *d*_eff_ = 2*n*_eff_ *d,* in which *d* is the physical thickness of the layer.^15,62–64^ In a poorly ordered structure, the presence of misoriented lamellae and diffused domains resulted in a poorly defined *d*_eff_ due to diminished structural coherence. To this end, SAXS measurements of the c-DB_2800_/l-DB_2800_ binary blend by increasing the weight fraction of linear diblock copolymer (*w*_l‑DB_) were performed and are presented in Figure 4A. We denote c-DB_2800_/l-DB_2800_ binary blends as c/l-DB_2800_(*x*), where x represents *w*_l‑DB_. Adding the l-DB_2800_ swelled the lamellae of c-DB_2800_, leading to the formation of larger domain sizes of the symmetric lamellar, as the position of the principal peaks, q*, shifted toward lower *q* values.^65^ When *w*_l‑DB_ was increased, the higher-order Bragg peaks at *q*/q* = 3, 5, 7, and 9, which were observed in neat c-DB_2800_, gradually diminished and eventually disappeared at a high *w*_l‑DB_ loading of 60 wt %. Next, we measured the UV–vis spectra of the c-DB_2800_/l-DB_2800_ binary blend with various *w*_l‑DB_ fractions (0.1–0.6) while keeping the thickness of the film constant (0.24 ± 0.02 mm). As depicted in Figure 4B,C, by increasing *w*_l‑DB_ in the c-DB_2800_ photonic film, a minimal shift of λ_max_ toward a red-shift region was observed, with no apparent color change in the blend film. The plot of a measurement of the full width at half-maximum of the peak relative to the peak maximum (Δλ/λ_max_) as a function of the added l-DB_2800_ exhibited significant peak broadening and lower peak intensity as *w*_l‑DB_ was increased. Blending 60 wt % of l-DB_2800_ into c-DB_2800_ bleached out its original color, yielding a macroscopically opaque bulk film just like neat l-DB_2800_, as depicted in Figure 4B.

**Figure 4:**
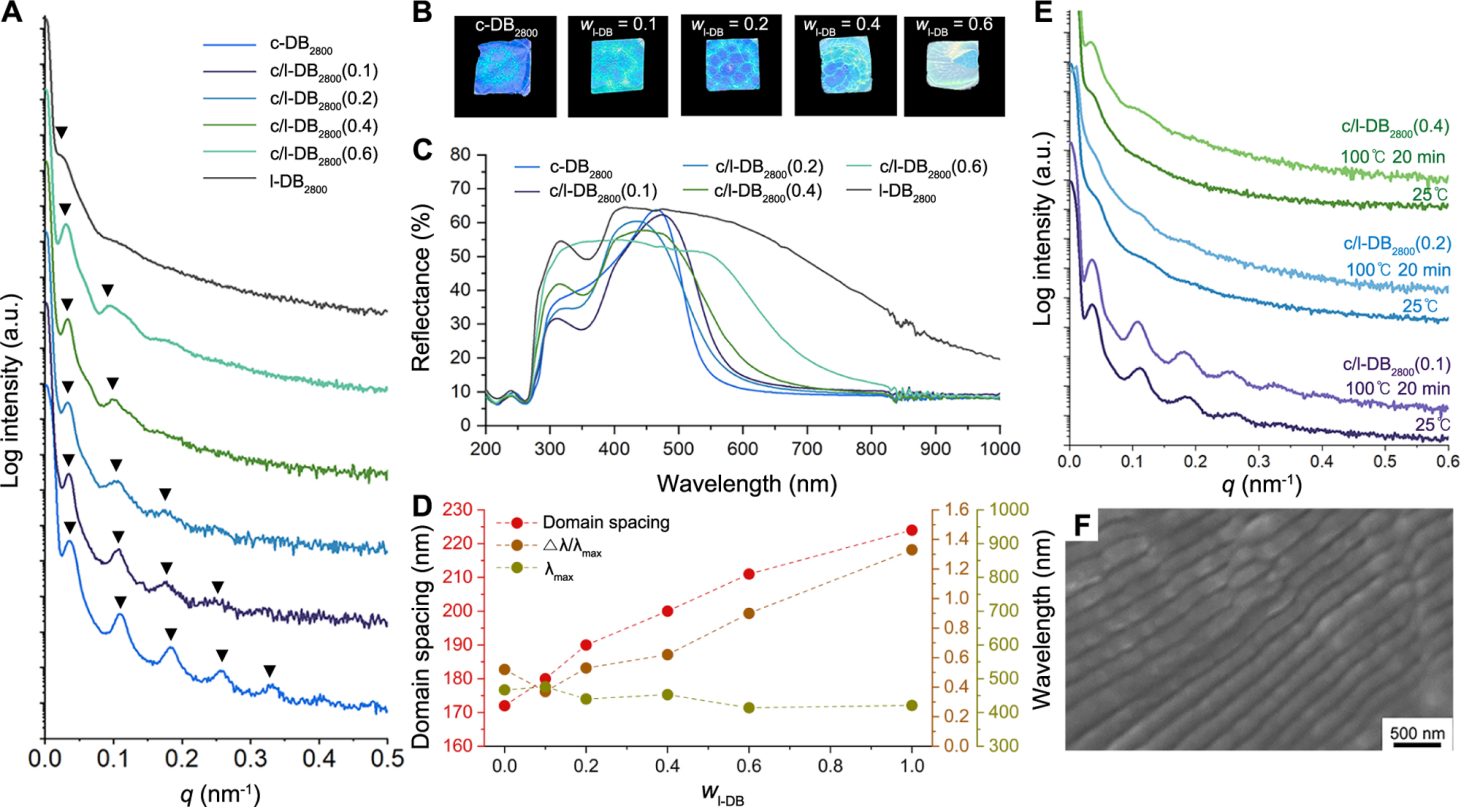
(A) SAXS profiles of c-DB_2800_, l-DB_2800_, and c/l-DB_2800_ blends with *w*_l‑DB_ = 0.1–0.6. (B) Photographs of bulk films of c-DB_2800_ and c/l-DB_2800_ with *w*_l‑DB_ = 0.1–0.6. (C) Plots of reflectance as a function of wavelength for c-DB_2800_, l-DB_2800_, and c/l-DB_2800_ blends with *w*_l‑DB_ = 0.1–0.6. (D) Plots of domain spacing of a phase-separated structure, Δλ/λ_max_, and λ_max_ as a function of *w*_l‑DB_. (E) In situ time-resolved SAXS patterns for c/l-DB_2800_(0.1), c/l-DB_2800_(0.2), and c/l-DB_2800_(0.4). The bulk samples were prepared by solvent casting from THF, followed by evaporation at RT. (F) Cross-sectional SEM image of neat c-DB_2800_ film.

The concurrent deterioration in photonic properties and phase behavior with increasing *w*_l‑DB_, as shown in Figure 4D, suggested that changes in λ_max_ and Δλ/λ_max_ were closely correlated with the structural order of the lamellar structure, including the purity of the domain and the lateral order of the phase-separated nanostructure, which will primarily influence *d*_eff_. In detail, the broadening of Δλ/λ_max_, manifested as a muted or opaque white appearance, was likely induced by the loss of long-range periodicity. This structural collapse, evidenced by the apparent trend of disappearance of high-order reflections, suggested a disruption in the coherence of reflections occurred due to randomly oriented lamellar grains. Owing to the limiting factors, the c-DB_2800_/l-DB_2800_ binary blend exhibited the muted bluish green or even opaque white color, although the lamellar periodicities of the blends were swollen from 110% for c/l-DB_2800_(0.2) to 123% for c/l-DB_2800_(0.6), relative to that of the neat c-DB_2800_. In the time-resolved SAXS profile of the binary blends, only c/l-DB_2800_(0.1) can form a highly ordered lamellar structure, while a marginal increase in the scattering peak intensity and limited development of the higher-order scattering function of c/l-DB_2800_(0.2) and (0.4) were induced upon annealing (Figure 4E). This observation indicated that the accumulation of chain entanglement between linear/linear and linear/cyclic chains in the blend increased the kinetic barrier for self-assembly, thereby suppressing the phase separation into well-defined phase-separated structures.^18,66^ Consistent with these findings, a correlation was observed between domain spacing and optical properties in c/l-DB_2800_(0.1), where an increase in the domain spacing to 180 nm corresponded to an increase in λ_max_ to 476 nm. This shift is attributed to light reflection through a well-defined lamellar structure, showing cyan color with a relatively narrower reflectance peak compared to the less ordered blends. This trend strongly supports the idea that *d*_eff_ may not increase proportionally to physical lamellar thickness within poorly defined lamellar structures that contain misoriented lamellar grains with low phase purity. Overall, these data are consistent with our interpretation that the well-defined lamellar array of c-DB_2800_, as determined by scanning electron microscopy (SEM) (Figure 4F), facilitated by suppressed chain entanglement and enhanced intermolecular interaction from compact packing contributes to the production of photonic films exhibiting saturated structural color.

Driven by the efficient phase-separation behavior of the cyclic topology, blending with medium-MW CPs facilitated a red-shifted structural coloration in films, as shown in Figure 5. In detail, c-PDA-*b*-PMMA (c-DM_120_, *M*_w_ = 74.7 kDa) and c-PBnMA (c-B_120_, *M*_w_ = 105.6 kDa) were obtained via LPP, respectively, as CPs corresponding to each block in c-DB_2800_ (Figures S20–S24 and S26 and Table S2). All ternary polymer blends contained a mixture of c-DM_120_ and c-B_120_ CP additives with equal amounts by weight in c-DB_2800._ The ternary blends were denoted as c-DB_2800_/c-DM_120_(*x*)/c-B_120_(*x*), where x represents the added weight fraction in the films. By increasing the *w*_c‑DM+c‑B_ (0.2–0.4), λ_max_ peaks were shifted continuously to 517 nm for 20 wt %, 546 nm for 30 wt %, and 608 nm for 40 wt % with minimal increments of the Δλ/λ_max_ in the bulk films with an average thickness of 0.28 ± 0.08 mm (Figure 5C). Accordingly, bluish green, yellowish green, and orange photonic films were obtained, as depicted in Figure 5B. The change in photonic properties corresponds to the distinctive phase separation behavior of the ternary blends, as shown in the SAXS profile (Figure 5A). As *w*_c‑DM+c‑B_ was increased, the primary peak position shifted toward the lower *q* values, indicating that the swollen lamellar periodicity from 181 to 211 nm of that of the neat c-DB_2800._ The substantial increase in the lamellar size upon blending implied that the medium-MW CP preferentially accumulated at the interior of the phase-separated domain rather than diffusing toward the domain interface.^67,68^ Upon adding *w*_c‑DM+c‑B_ (0.4) to the ternary blend, the narrow and intense scattering reflections were preserved, followed by a higher-order scattering function at *q*/q* = 7. Owing to the high structural ordering of the cyclic ternary blended film, the *d*_eff_ increased depending on swelling of physical lamellar thickness within the well-defined lamellar structure, shifting to longer reflected wavelengths (Figure 5D). Further increasing of *w*_c‑DM+c‑B_ to 0.5 showed only a marginal increase of λ_max_ with periodicity, possibly due to a lack of long-range order and lower purity of the phase-separated domains of the lamellar structure (Figures S27 and S28).

**Figure 5:**
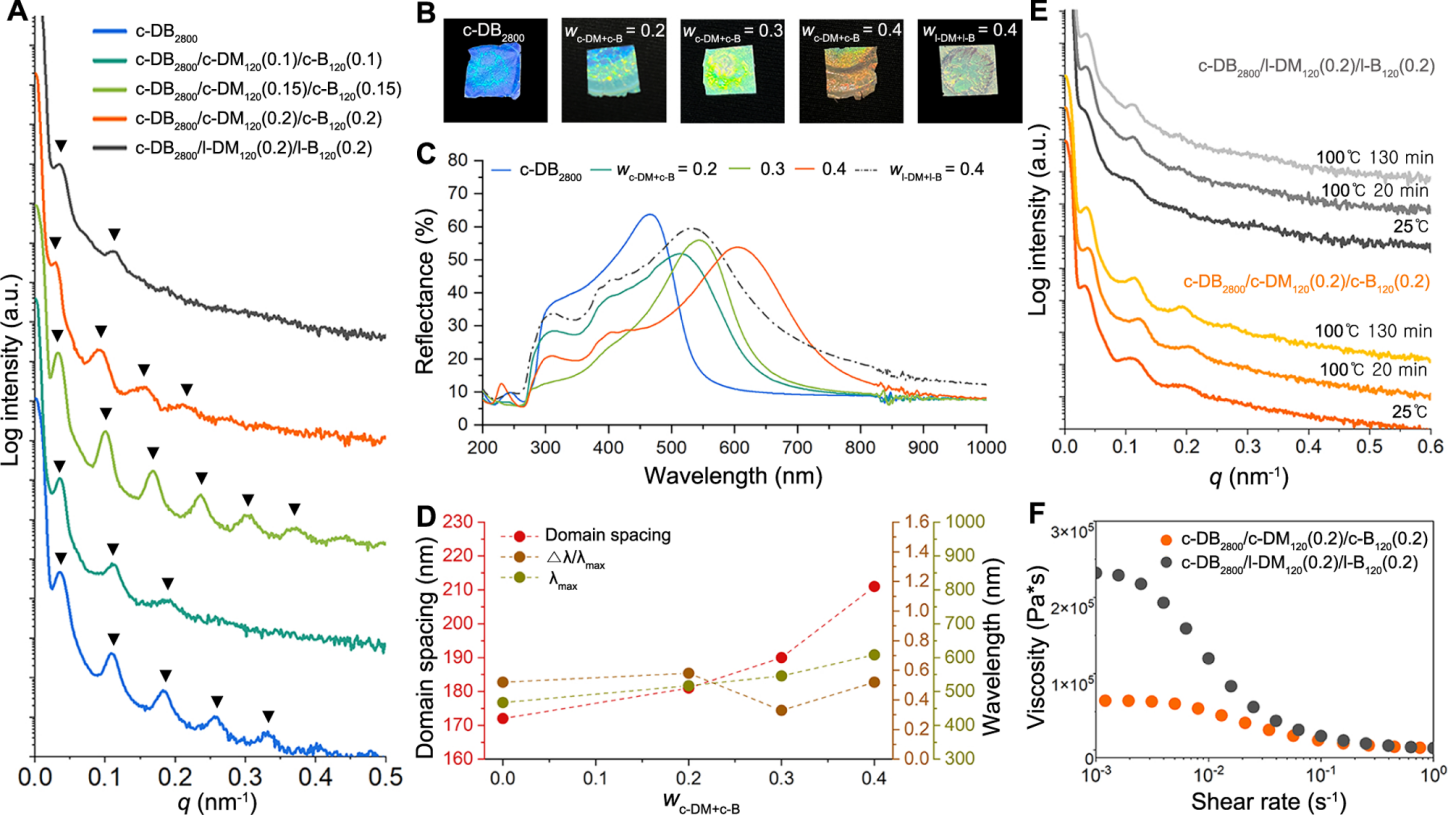
(A) SAXS profiles of neat c-DB_2800_, c-DB_2800_/c-DM_120_/c-B_120_ with *w*_c‑DM+c‑B_ (0.2–0.4), and c-DB_2800_/l-DM_120_(0.2)/l-B_120_(0.2). (B) Photographs of bulk films of c-DB_2800_, c-DB_2800_/c-DM_120_/c-B_120_ with *w*_c‑DM+c‑B_ (0.2–0.4), and c-DB_2800_/l-DM_120_(0.2)/l-B_120_(0.2). (C) Plots of reflectance as a function of wavelength of c-DB_2800_, c-DB_2800_/c-DM_120_/c-B_120_ with *w*_c‑DM+c‑B_ (0.2–0.4), and c-DB_2800_/l-DM_120_(0.2)/l-B_120_(0.2). (D) Plots of domain spacing of the phase-separated structure of ternary blend containing cyclic additives, Δλ/λ_max_, and λ_max_ as a function of *w*_c‑DM+c‑B_ (0–0.4). (E) In situ time-resolved SAXS patterns for c-DB_2800_/c-DM_120_(0.2)/c-B_120_(0.2) and c-DB_2800_/l-DM_120_(0.2)/l-B_120_(0.2). The bulk samples were prepared by solvent casting from THF, followed by evaporation at RT. (F) Zero shear viscosity of c-DB_2800_/c-DM_120_(0.2)/c-B_120_(0.2) and c-DB_2800_/l-DM_120_(0.2)/l-B_120_(0.2) at 160 °C.

When the scattering pattern of a ternary blend containing linear polymer additives (*M*_w, l‑B_ = 121.9 kDa and *M*_w, l‑DM_ = 65.8 kDa) with the same weight fraction of 0.4 was compared (Figures S20, S22–S23, and S25 and Table S2), c-DB_2800_/l-DM_120_(0.2)/l-B_120_(0.2) exhibited poorer lamellar ordering with broader and less intense scattering peaks. Correspondingly, the vibrant orange structural color observed in c-DB_2800_/c-DM_120_(0.2)/c-B_120_(0.2) was substantially diminished in the c-DB_2800_/l-DM_120_(0.2)/l-B_120_(0.2) system, being opaque in the film. The in situ time-resolved SAXS measurement revealed that the blend with CP additives self-assembled in a faster manner than did the addition of linear polymers (Figure 5E). In RT-evaporated c-DB_2800_/c-DM_120_(0.2)/c-B_120_(0.2) bulk films, the distinct peaks appeared with scattering reflections at *q*/q* = 1, 3, and 5. After annealing at 100 °C for 20 and 130 min, the sharper and more intense scattering peaks were developed, indicating the growth of purer domains with well-defined interfaces. However, the broader and lower intensity of Bragg peaks at *q*/q* = 1 and 3 were observed in as-prepared c-DB_2800_/l-DM_120_(0.2)/l-B_120_(0.2) bulk at 25 °C. Upon annealing at 100 °C, a gradual increase in peak intensity was observed after 20 min, but the overall scattering pattern remained largely unchanged with prolonged annealing. We reasoned that the more rapid chain rearrangement with CP additives than the linear one was attributed to the lower viscosity of the ternary blend, which resulted from the suppressed entanglement between c-DB_2800_ and CP additives (Figure 5F). Overall, these data are consistent with our findings that the well-defined lamellar array, facilitated by suppressed chain entanglement and reduced conformation freedom of the self-assembly of CDCs, contributes to producing photonic films reflecting tunable, vivid structural colors.

## Conclusions

This work has demonstrated the effective self-assembly of the CDC system conveniently synthesized on scale via LPP into PCs. Thanks to the compounded thermodynamic (biased MAD affinity toward acrylate DA over methacrylate BnMA) and kinetic (higher propagation rate of DA over BnMA) sequence control, LPP of one-pot comonomer mixtures of DA and BnMA enabled strictly sequential incorporation of DA and then BnMA, producing the well-resolved PDA-*b*-PBnMA block sequence absent of tapered regions. Subsequent spatiotemporally controlled conjugate addition of the ES-derived initiating chain end and the anionic enolate terminus produced the CDC with HMW at scale (20 g). The quantitative transformation of the linear to cyclic topology during LPP was obtained, as monitored by NMR, and the formation of the cyclic topology was determined by the observed significant shrinkage of *R*_g_ (*R*_g,c‑DB_^2^/*R*_g,l‑DB_^2^ = ca. 0.65), as well as the reduction of intrinsic viscosity in solution ([η]_c‑DB_/[η]_l‑DB_ = ca. 0.65) and of melt viscosity (η_0,c‑DB_/η_0,l‑DB_ = ca. 0.57), all indicative of a compact CDC structure.

The resulting CDC self-assembled into a well-defined lamella with a domain spacing of 172 nm, yielding a vibrant, blue photonic film. By blending it with medium-MW CP additives corresponding to the CDC components, the ternary blends exhibited a swollen domain spacing from 181 to 211 nm, allowing precise tuning of the reflected wavelength peak across a visible spectrum range from 467 to 608 nm. In addition, the cyclic topology exhibited a superior photonic response compared with its linear counterpart by revealing a difference in phase separation behavior correlated to coherent reflectance. Faster chain dynamics of the less entangled cyclic topology, determined by in situ time-resolved SAXS, favored spontaneous self-assembly into a highly ordered lamellar. The well-defined *d*_eff_, resulting from the long-range ordered lamellar with highly segregated domains, afforded shifts of λ_max_ corresponding to the increased lamellar periodicity while maintaining narrow Δλ/λ_max_, showing vibrant blue to green to orange structural colors.

Overall, the results reported herein demonstrate that (a) LPP is a uniquely effective method to synthesize well-resolved, HMW linear and cyclic block copolymers from a one-pot mixture of comonomers; (b) the CDC system is notably superior to its linear counterpart in the context of applications in producing structural colors of block copolymer-based PCs, thanks to both thermodynamic (more compact packing) and kinetic (less entangled cyclic chains) gains that not only control but also accelerate the self-assembling process into highly ordered nanostructures for intensely selective reflection and improved optical appearance or colors; and (c) blending of the CDC with constituent CPs further enhances CDC’s photonic responses, offering a convenient strategy to tune the structural colors of CDC-based photonic polymeric materials.

## Supplementary Material



## Notes

Synthetic protocol and characterization data for CDCs and linear diblock copolymers by NMR, SEC, DSC, and TEM and characterization data for self-assembled blends by SAXS analysis and UV–vis spectroscopy (PDF)
