# Supplementary material for: Structural Colors of Cyclic Diblock Copolymers Enabled by Topology-Driven Structural Ordering and Tuned by Blending with Constituent Cyclic Polymers
Source: J Am Chem Soc. 2026 Feb 27;148(12):12870–9. doi: 10.1021/jacs.5c21376 (PMC13047683; doi:10.1021/jacs.5c21376)
Supplement: Supplementary file 1 [file ja5c21376_si_001.pdf]

Supporting Information

Structural Colors of Cyclic Diblock Copolymers Enabled by Topology-Driven Structural Ordering and Tuned by Blending with Constituent Cyclic Polymers

Jiyun Nam and Eugene Y.-X. Chen*

Department of Chemistry, Colorado State University; Fort Collins, CO 80523, United States

*To whom should be addressed: eugene.chen@colostate.edu

This information includes:

Materials and Methods

Experimental Details

Supporting Figures S1-S28

Supporting Tables S1-S2

Supporting References

Materials and Methods

Materials

Monomers dodecyl acrylate (DA), benzyl methacrylate (BnMA), and ethyl sorbate (ES) were all purchased from TCI, dried under CaH_2, and vacuum-distilled prior to storage at -30 °C inside a freezer of an inert glovebox. *N*-Heterocyclic carbene (NHC) 1,3-di-tert-butylimidazol-2ylidene (I^tBu) was purchased from TCI and used as received. Tricyclohexylphosphine (PCy_3) and basic alumina were purchased from Sigma-Aldrich and used as received. HPLC grade toluene and tetrahydrofuran (THF) were purchased from Sigma-Aldrich. Toluene was dried over NaK alloy and filtered through a 0.2 µm nylon membrane filter prior to use. Methylaluminum bis(2,6-di-tert-butyl-4-methylphenoxide) (MAD) was synthesized according to literature procedures.[1]

Methods

NMR spectroscopy. Monomer conversion, characterization of catalysts and polymers, polymerization kinetics, and polymer block architecture were determined by ^{1}H NMR and ^{13}C NMR experiments using either a Varian Inova 400 MHz (FT 400 MHz ^{1}H; 100 MHz ^{13}C) or a Bruker AVIII 400 MHz spectrometer (400 MHz ^{1}H; 100 MHz ^{13}C). Variable-Temperature ^{1}H NMR experiments were performed to obtain K_{eq} values using an Agilent Inova 500 MHz spectrometer (500 MHz ^{1}H NMR). Chemical shifts were referenced to internal solvent resonances at 7.26 ($CHCl_3$) and 7.16 (C_6D_6) ppm.

Size exclusion chromatography (SEC). Measurements of polymer weight-average molecular weight (M_w), number-average molecular weight (M_n), and molecular weight dispersity ($Đ = M_w/M_n$) were performed via size-exclusion chromatography (SEC). The SEC instrument consisted of an

Agilent HPLC system equipped with one guard column and three PLgel 5 μm mixed-C gel permeation columns unless indicated otherwise and coupled with a Wyatt DAWN HELEOS II multi (18)-angle light scattering detector and a Wyatt Optilab TrEX dRI detector; the analysis was performed at 40 °C using chloroform as the eluent at a flow rate of 1.0 mL min^{-1}, using Wyatt ASTRA 7.1.2 molecular weight characterization software. All polymer solutions were prepared at the target concentration (~ 2 mg mL^{-1}), stirred for 24 h, and then filtered before analysis.

Differential scanning calorimetry (DSC). DSC was performed on dried polymer samples (4 mg) on an Auto Q20 (TA Instrument). All DSC plots represent the data obtained from a second heating scan after the thermal history was removed on the first heating scan (5 °C min^{-1}). The second heating scan was performed at a heating rate of 5 °C min^{-1} to 100 °C, following a cooling scan at a rate of 5 °C min^{-1} to -80 °C. Data analysis was performed using the Universal Analysis software (TA Instruments).

Rheology. Viscosity experiments by rheology were performed on dried linear diblock copolymers (LDC) and cyclic diblock copolymers (CDC) melts prepared by heated compression molding at 195 °C (between two steel plates, a 38.1 × 12.7 × 1 mm steel mold, and non-stick Teflon sheets) inside a Carver Bench Top Laboratory Press (Model 4386). In case of CDC/cyclic polymers (CP) and CDC/linear polymers (LP), blend melts were prepared at 160 °C. Small circular-cut (8 mm diameter) samples were loaded between two 8 mm steel electrically heated platen (EHP) loading discs within a Discovery Series HR-2 (Hybrid Rheometer) (TA Instruments) under nitrogen gas flow (30 psi) connected to the TRIOS software (TA Instruments). Viscosity was studied under the flow testing option and amplitude setting. Experiments were run at 195 °C for c-DB_{2800}, and l-DB_{2800}, or 160 °C for c-DB_{2800}/c-DM_{120}(0.2)/c-B_{120}(0.2) and c-DB_{2800}/l-DM_{120}(0.2)/l-B_{120}(0.2)

with shear rates varying between 10^{-3} and 10^{0} rad/*s*. The axial force was controlled within a negligible ± 0.1 *N* to prevent non-frictional forces.

X-ray scattering. Small-angle X-ray scattering (SAXS) and wide-angle X-ray scattering (WAXS) were performed with Xenocs Xeuss 3.0 SAXS/WAXS. The X-ray beam energy was 8,048 keV (Cu Kα, 1.54 Å) with a beam size of 0.7 (horizontal) × 0.7 (vertical) mm^2 for the slits closest to the sample. The images were taken with an Eiger2 R 1 M (Dectris) area detector comprising (1,028 × 1,062) pixels with a pixel size of 75 μm^2 in transmission geometry. The sample-to-detector distance was 1800 mm for SAXS and 42.5 mm for WAXS downstream of the sample. Silver behenate (AgBeh) for SAXS and lanthanum hexaboride (LaB_6) for WAXS were used as the standard to calibrate the sample-to-detector distance. The two-dimensional scattering patterns were azimuthally integrated to afford one-dimensional profiles presented as scattering vector *(q*) versus scattering intensity, where the magnitude of the scattering vector is calculated with $q = (4\pi/\lambda)\sin(\theta/2)$. Domain spacing (*d*) was obtained from the position of the principal scattering (q^*) using the relationship $d = 2\pi/q^*$. The samples were prepared by THF solution (30 mg mL^{-1}) to be evaporated for 3 days at room temperature (RT), then annealed at 120 °C for 16 h. Samples for time-resolved SAXS analysis were prepared by casting from THF, followed by drying 16 h at RT under vacuum. Measurements were conducted on the resulting as-prepared bulk films.

Transmission electron microscopy (TEM). The specimens were prepared with a polymer concentration of 0.1 mg mL^{-1} in toluene. Thin films of c-DB_{2800} were prepared by drop-casting onto a 200 mesh TEM grid. The grid was left to dry in RT for 16 h before placing it on the microscope. The TEM imaging was done at 200 kV using a JEOL 2100F transmission electron microscope.

Scanning electron microscopy (SEM). SEM was performed using a JEOL IT800HL microscope equipped with a high-brightness cold field emission gun (CFEG) and upper secondary electron detector, operated at an accelerating voltage of 5-10 kV. Prior to cross-sectional SEM imaging, the cryo-fractured bulk film was sputter-coated with a 10 nm layer of Cr.

UV-vis spectrometer. Reflection measurements were performed on a Shimadzu UV-3600i UV−vis/NIR spectrophotometer, equipped with MPC-603A integrating sphere accessory, using the standard wide-open sampling port. The samples were scanned from 1000 nm to 200 nm at a rate of 0.5 nm s^{-1} with a data interval of 0.5 nm. The detector crossover from InGaAs to PMT was set at 860 nm.

Experimental Details

Characterization of DA/BnMA/MAD equilibrium

In toluene-d_8, 0.080 M stock solutions of DA, BnMA, and MAD were prepared. Then, in four J-Young type NMR tubes, the following samples were prepared: (**1**) MAD alone 0.080 M in toluene; (**2**) MAD (0.30 mL of 0.080 M) + DA (0.30 mL of 0.080 M); (**3**) MAD (0.30 mL of 0.080 M) + BnMA (0.30 mL of 0.080 M); (**4**) MAD (0.20 mL of 0.080 M) + DA (0.20 mL of 0.080 M) + BnMA (0.20 mL of 0.080 M). Each sample was sealed and analyzed by ^{1}H NMR at -15 °C.

Preparation of ES/I*t*Bu/MAD mixture

Individual 0.3 M stock solutions of I^{t}Bu, MAD, and ES were prepared in toluene. Then, 0.3 mL of the ES solution was added to 0.3 mL of the MAD solution, giving a brilliant orange color. Then, 0.6 mL of the combined MAD/ES solution (now only 0.15 M) was added to 0.30 mL of I^{t}Bu solution dropwise over 1 min, generating the titled mixture at a theoretical concentration of 0.1 M.

The concentrations of the stock solutions were diluted with toluene to 0.01 M so that the final mixture would correspond to a quantity of initiator appropriate for each particular reaction.

Preparation of CDC/LDC, CDC/CP, and CDC/LP blends

Blends were prepared by dissolving c-DB_{2800} and l-DB_{2800} with the designated weight fraction in benzene to generate stock solutions. Then, the mixture in 5 ml scintillation vials was flash frozen by submerging the vials in liquid nitrogen. Once fully frozen, the samples were subjected to vacuum drying using a Schlenk line, yielding fluffy white powders composed of homogenous mixtures of all polymer components. The blends of c-DB_{2800}/c-DM_{120}/c-B_{120} and of c-DB_{2800}/l-DM_{120}/l-B_{120} were prepared using the same procedure, following the specified weight fraction of each component.

Polymerization Procedure

Polymerization to LDCs. Synthesis of l-DB_{2800} is given as a representative example. In a 20 mL vial equipped with a stir bar, MAD (0.015g, 0.0312 mmol) was weighed out precisely and dissolved in 0.3 mL of toluene. Then DA (0.27 g, 1.119 mmol) and BnMA (0.35 g, 1.961 mmol) were added to the MAD solution to make a bright yellow solution. After dissolving the polymerization mixture into toluene (5.0 mL), a stock solution of PCy_3 (0.0003 g, 0.0011 mmol) was injected into the solution to initiate the polymerization. The reaction mixture became clear after 10 min at RT. Methanol (1 mL) spiked with 100 ppm benzoic acid was added to quench the polymerization mixture. Small aliquots were withdrawn for obtaining quantitative conversion data by ^1H NMR analysis. The remaining reaction mixture was then concentrated and precipitated into methanol (200 mL). The polymeric product was recovered by filtration and dried under vacuum

at 40 °C for 16 h. All the LDCs and LPs were synthesized following the protocol described above. Table S1 summarizes detailed information for the LPP results reported in this study.

Polymerization to l-BDB$_{2800}$. In a 20 mL vial equipped with a stir bar, MAD (0.0444 g, 0.0924 mmol) was weighed out precisely and dissolved in 0.3 mL of toluene. Then BnMA (0.51 g, 2.890 mmol) was added to the MAD solution to make a bright yellow solution. After dissolving the mixture into toluene (5.8 mL), a stock solution of PCy_3 (0.0009 g, 0.0032 mmol) was injected into the solution to initiate the polymerization. The polymerization solution was stirred for 5 min at RT. Then the mixture of DA (0.79 g, 3.295 mmol) and BnMA (0.51 g, 2.890 mmol) were added to the polymerization solution and became clear after 10 min at RT. Methanol (1 mL) spiked with 100 ppm benzoic acid was added to quench the polymerization mixture. Small aliquots were withdrawn for obtaining quantitative conversion data by ^{1}H NMR analysis. The remaining reaction mixture was then concentrated and precipitated into methanol (200 mL). The polymeric product was recovered by filtration and dried under vacuum at 40 °C for 16 h. Table S1 summarizes detailed information for the LPP results reported in this study.

Polymerization to CDCs. Synthesis of c-DB$_{2800}$ is given as a representative example. In a 200 mL Schlenk flask equipped with a stir bar, MAD (0.533 g, 1.110 mmol) was weighed out precisely and dissolved in 1.0 mL of toluene. Then DA (9.02 g, 37.52 mmol) and BnMA (11.59 g, 65.79 mmol) were added to the MAD solution to make a bright yellow solution. After dissolving the polymerization mixture into toluene (172 mL), a stock solution of ES/I^{t}Bu/MAD mixture (0.0369 mmol) was injected into the solution to initiate the polymerization. The reaction mixture became clear after 20 min at RT. Small aliquots were withdrawn for obtaining quantitative conversion data for ^{1}H NMR analysis. Unlike the linear analogue, a slight yellow tint remained in the reaction even

after full conversion. The reaction was allowed to stir for 3d following complete conversion to ensure complete cyclization. Then, 1 mL of methanol spiked with 100 ppm benzoic acid was added to quench the polymerization mixture. The remaining reaction mixture was then concentrated and precipitated into methanol (500 mL). The polymeric product was recovered by filtration and dried under vacuum at 40 °C for 16 h. All the CDCs and CPs were synthesized following the protocol described above. Table S1 summarizes detailed information for the LPP results in this study.

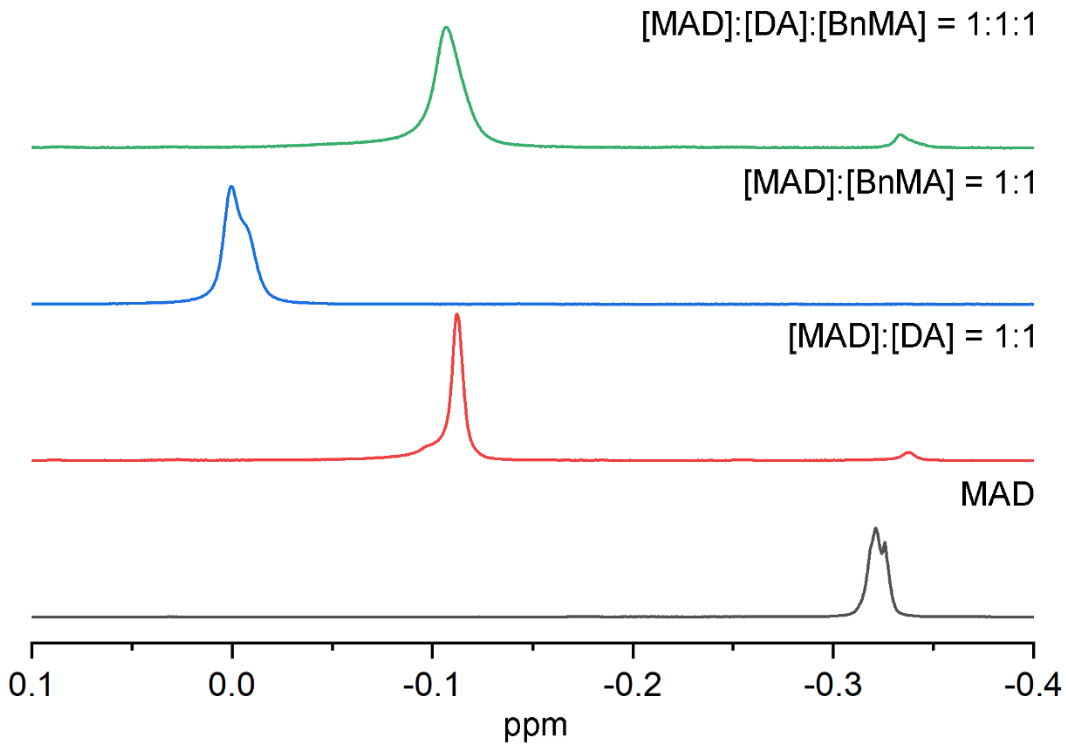

Figure S1. ^{1}H NMR spectral overlay (toluene-d_8, -15 °C) for the equilibrium study of DA vs BnMA affinity with MAD, using the Al-CH_3 shift as the dependent variable, MAD (black), [MAD]:[DA] = 1:1 (red), [MAD]:[BnMA] = 1:1 (blue), [MAD]:[DA]:[BnMA] = 1:1:1 (green).

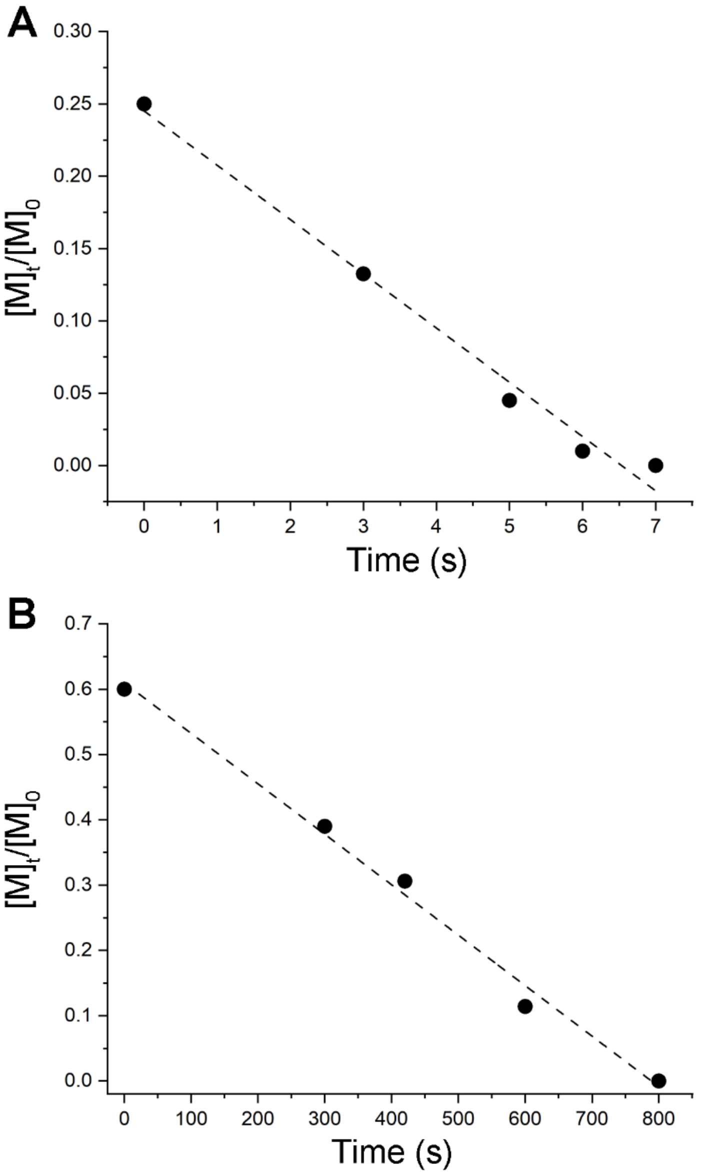

Figure S2. Zero-order kinetic plots for PDA polymerization ($[DA]_0$:$[MAD]_0$:$[ESIP]_0$ =1017:2:1) $[DA]_0$ = 0.25 M, $[MAD]_0$ = 0.00049 M, $[ESIP]_0$ = 0.00024 M; $k_{p,\ obs}$ = 1.28 × 10^5 $[M]\cdot s^{-1}\cdot[MAD]_t^{-1}\cdot[LB]_0^{-1}$. (A), and PBnMA polymerization ($[BnMA]_0$:$[MAD]_0$:$[ESIP]_0$ =1783:18:1) $[BnMA]_0$ = 0.6 M, $[MAD]_0$ = 0.011 M, $[ESIP]_0$ = 0.00036 M; $k_{p,\ obs}$ = 3.2× 10^2 $[M]\cdot s^{-1}\cdot[MAD]_t^{-1}\cdot[LB]_0^{-1}$ (B) in toluene at RT.

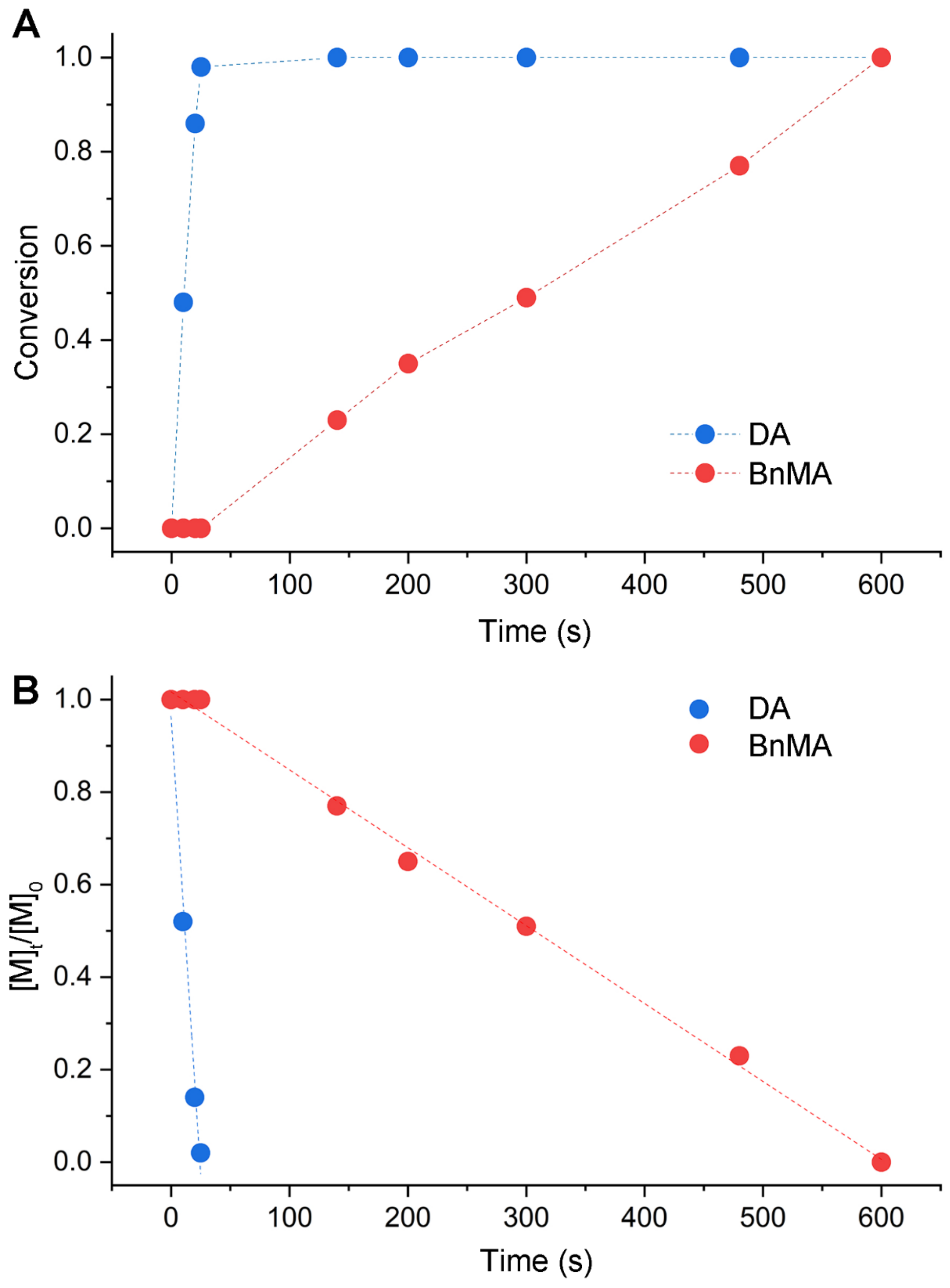

Figure S3. (A) DA (blue dots) and BnMA (red dots) conversion data during the LPP for the synthesis of l-DB$_{2800.}$ (B) Plot of zero-order kinetics ($[DA]_0$:$[BnMA]_0$:$[MAD]_0$:$[PCy_3]_0$ =1017:1783:28:1, $[M]_0$ = 0.6 M in toluene at RT).

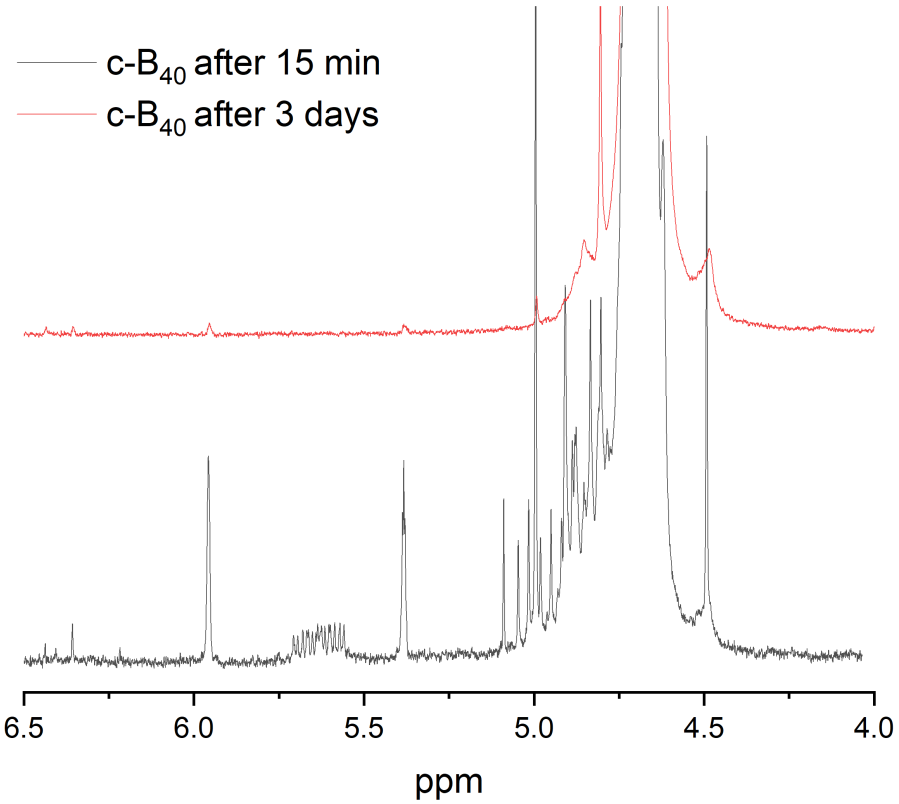

Figure S4. ^{1}H NMR spectra ($CDCl_3$) of c-B_{40} quenched after 15 min (black) and 3 days (red) during LPP, showing quantitative cyclization.

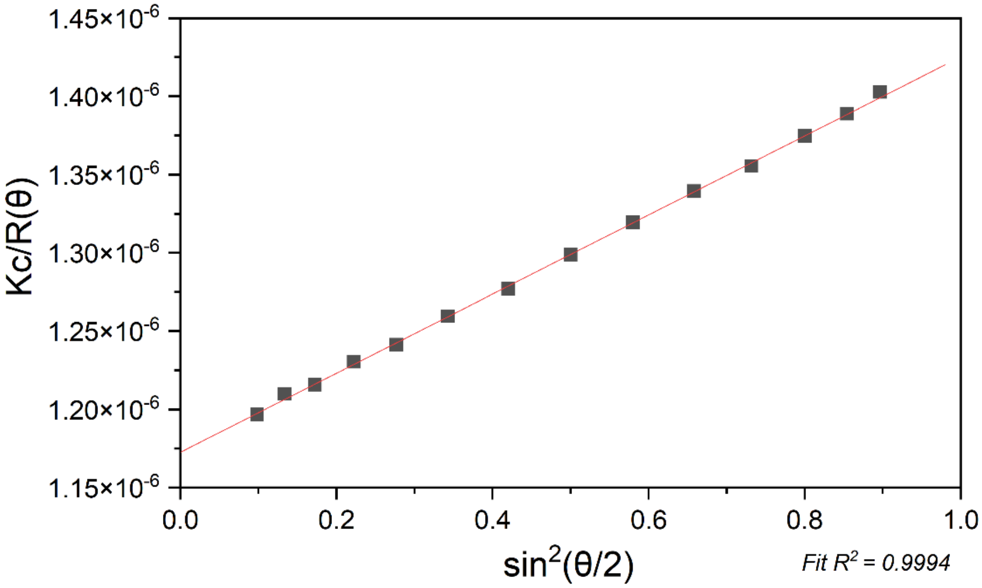

Figure S5. Kc/R(θ) plotted as a function of $\sin^2(\theta/2)$ for c-DB_{2800}, obtained from the angular dependence of the excess scattering for a single MALS-SEC elution slice at a polymer concentration of 2 mg mL^{-1} (estimated d*n*/d*c* from the elution slice = 0.0719 mL/g).

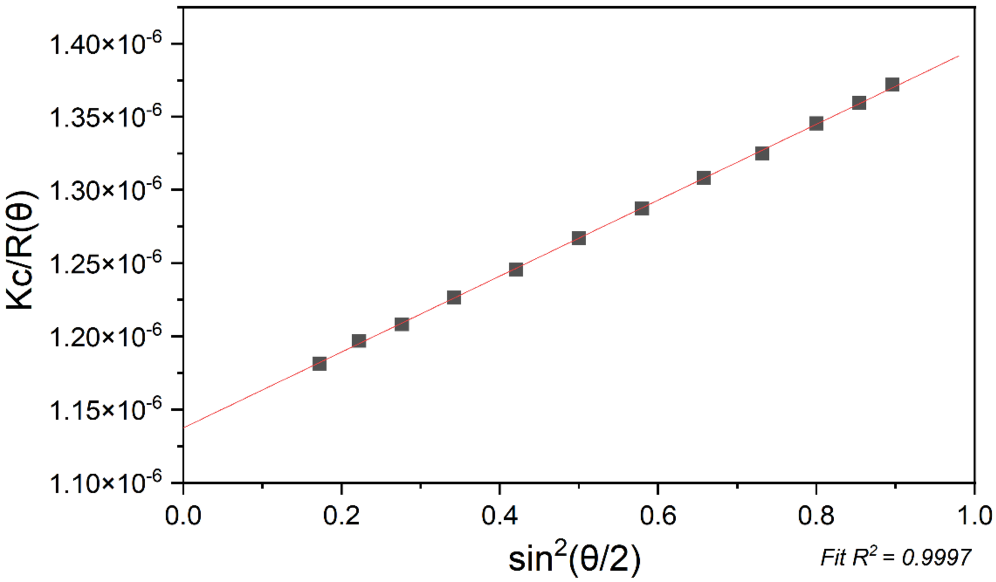

Figure S6. Kc/R(θ) plotted as a function of $\sin^2(\theta/2)$ for l-DB_{2800}, obtained from the angular dependence of the excess scattering for a single MALS-SEC elution slice at a polymer concentration of 2 mg mL^{-1} (estimated d*n*/d*c* from the elution slice = 0.0857 mL/g).

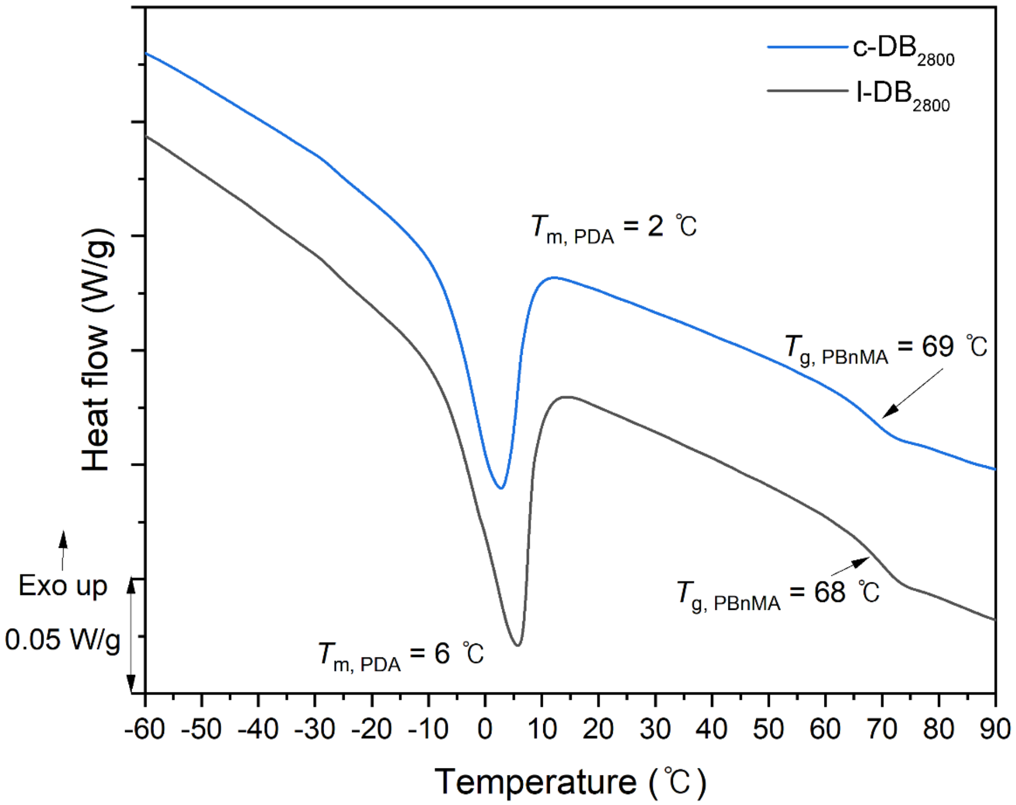

Figure S7. DSC traces (2nd heating scan) of c-DB_{2800} and l-DB_{2800}.

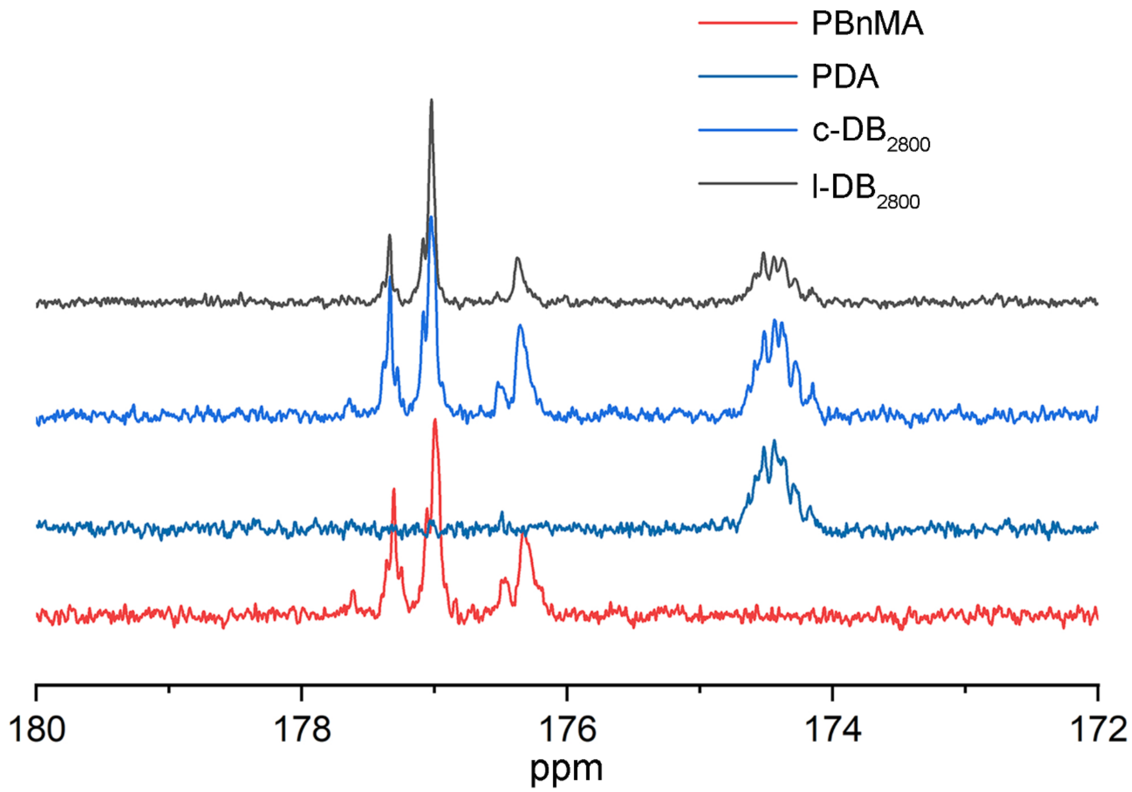

Figure S8. ^{13}C NMR spectral overlay ($CDCl_3$) in the carbonyl region of c-DB_{2800}, l-DB_{2800}, PBnMA, and PDA.

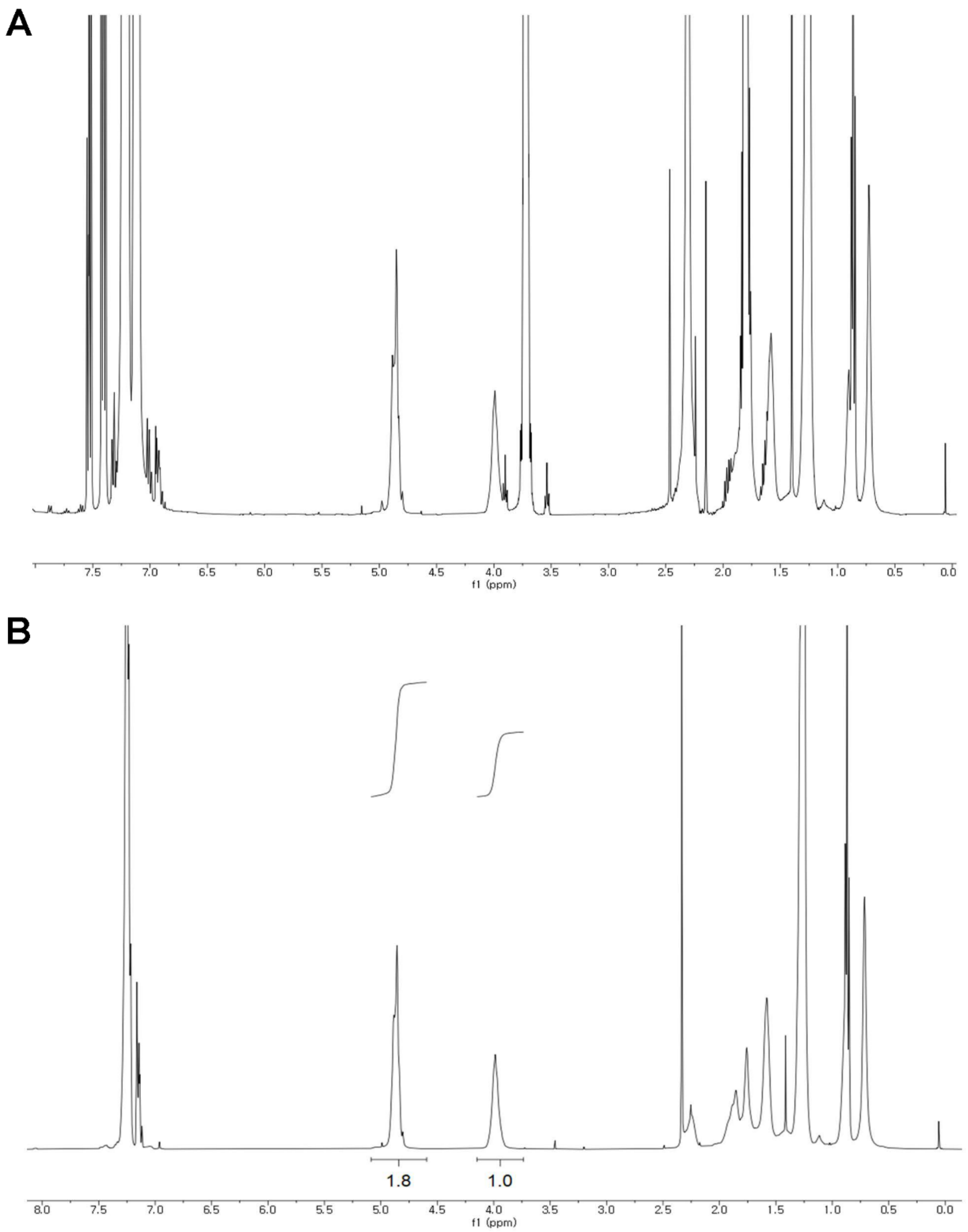

Figure S9. ^{1}H NMR spectra ($CDCl_3$) of quenched (A) and isolated (B) c-DB_{2800} with a theoretical comonomer incorporation of DA:BnMA =1017:1783, corresponding to the DA:BnMA integration ratio of 1.0:1.8 for DA alkoxy methylene protons at 3.98 ppm and BnMA alkoxy methylene protons at 4.80 ppm, respectively.

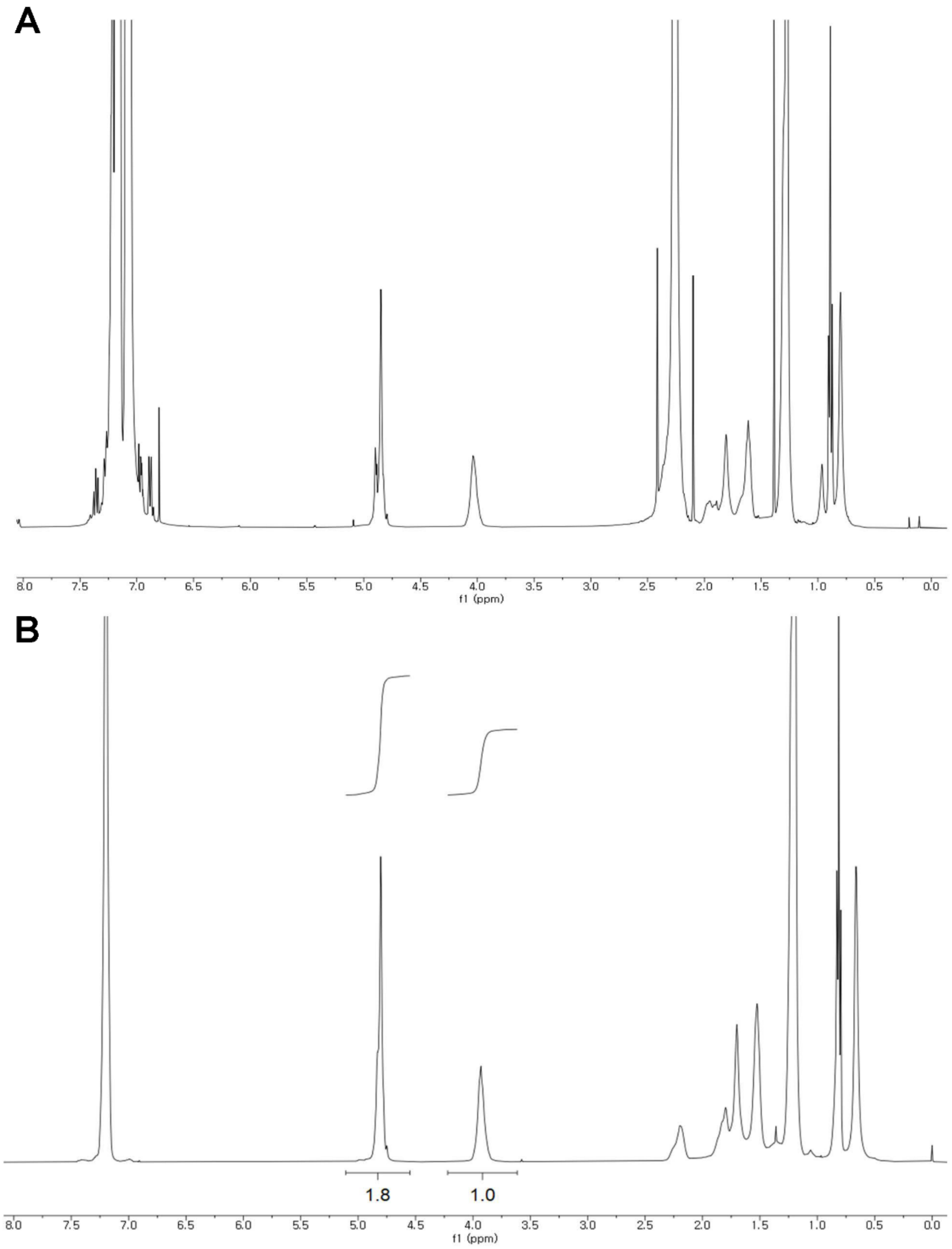

Figure S10. ^{1}H NMR spectra ($CDCl_3$) of quenched (A) and isolated (B) l-DB_{2800} with a theoretical comonomer incorporation of DA:BnMA =1017:1783, corresponding to the DA:BnMA integration ratio of 1.0:1.8 for DA alkoxy methylene protons at 3.98 ppm and BnMA alkoxy methylene protons at 4.80 ppm, respectively.

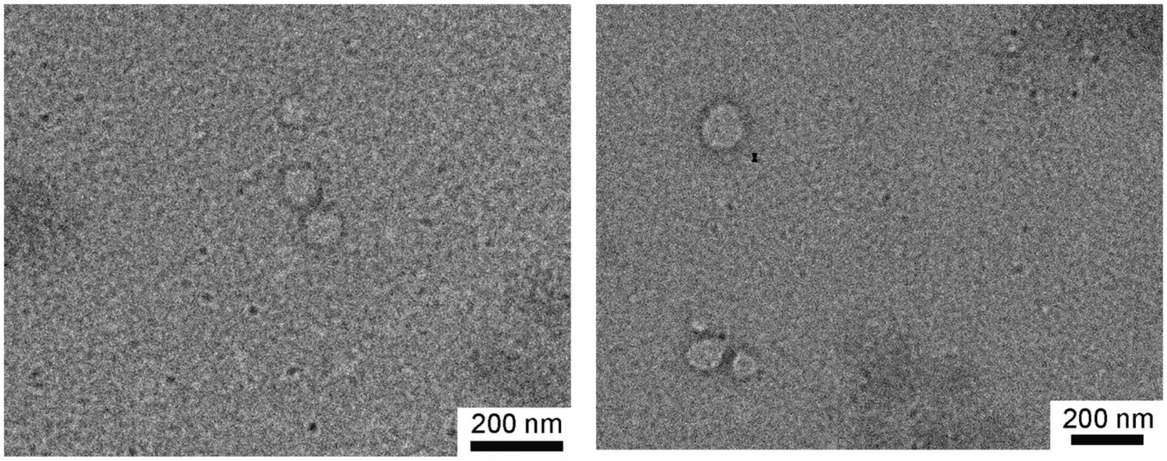

Figure S11. TEM images of c-DB_{2800} at low magnification. The specimen was prepared by polymer concentration of 0.1 mg mL^{-1} in toluene.

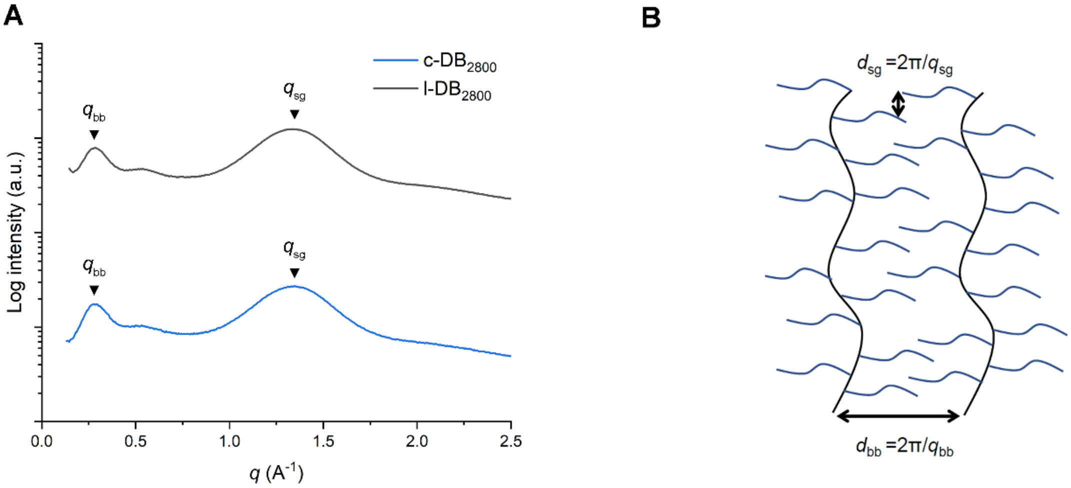

Figure S12. (A) WAXS profiles of l-DB_{2800}, and c-DB_{2800}. The low-q peak corresponds to the average inter-backbones correlation (q_{bb}), and the high-q feature corresponds to that of the side groups (q_{sg}). (B) Schematic illustration of the average d_{bb} and d_{sg} in the PDA block.

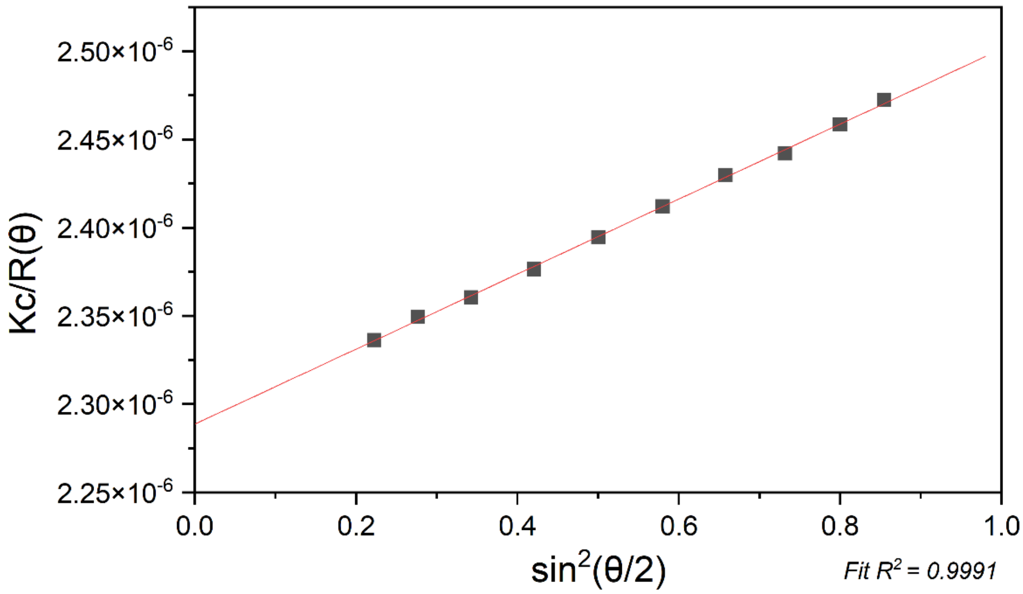

Figure S13. Kc/R(θ) plotted as a function of $\sin^2(\theta/2)$ for l-DB_{1400}, obtained from the angular dependence of the excess scattering for a single MALS-SEC elution slice at a polymer concentration of 2 mg mL^{-1} (estimated d*n*/d*c* from the elution slice = 0.0755 mL/g).

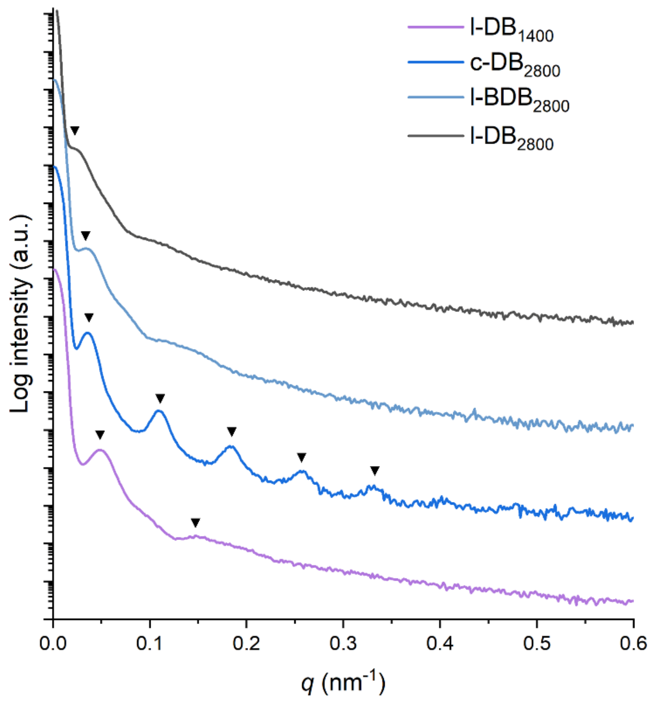

Figure S14. SAXS profiles of l-DB_{2800}, l-BDB_{2800}, c-DB_{2800}, and l-DB_{1400}.

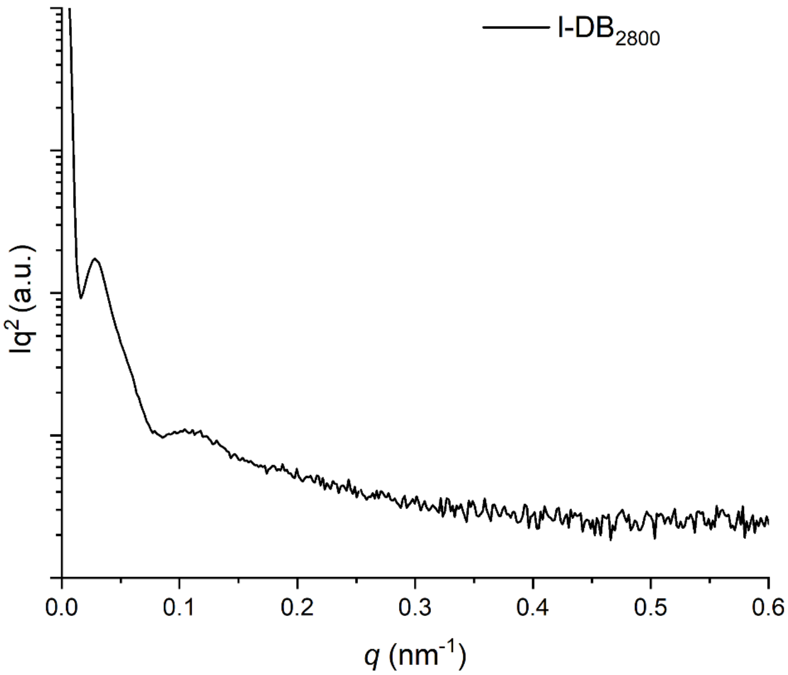

Figure S15. Lorentz-corrected SAXS profile of l-DB_{2800}.

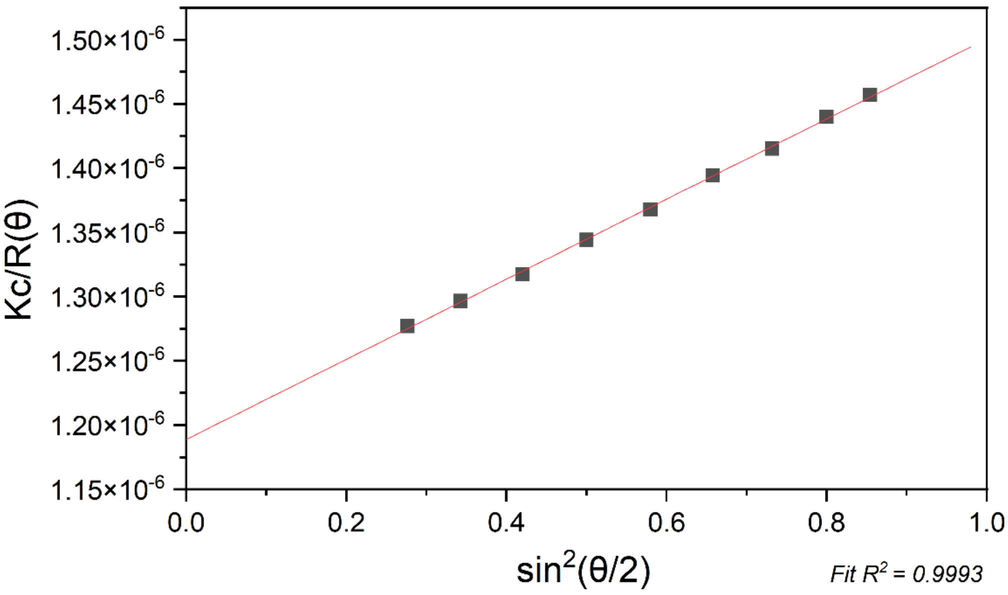

Figure S16. Kc/R(θ) plotted as a function of $\sin^2(\theta/2)$ for l-BDB_{2800}, obtained from the angular dependence of the excess scattering for a single MALS-SEC elution slice at a polymer concentration of 2 mg mL^{-1} (estimated d*n*/d*c* from the elution slice = 0.0755 mL/g).

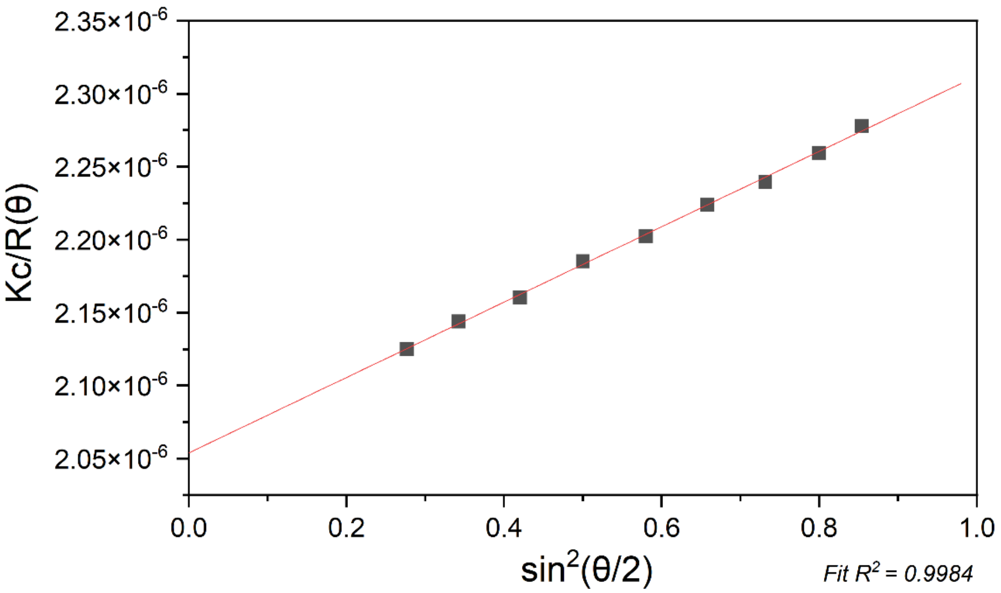

Figure S17. Kc/R(θ) plotted as a function of $\sin^2(\theta/2)$ for c-DB_{1400}, obtained from the angular dependence of the excess scattering for a single MALS-SEC elution slice at a polymer concentration of 2 mg mL^{-1}(estimated d*n*/d*c* from the elution slice = 0.0691 mL/g).

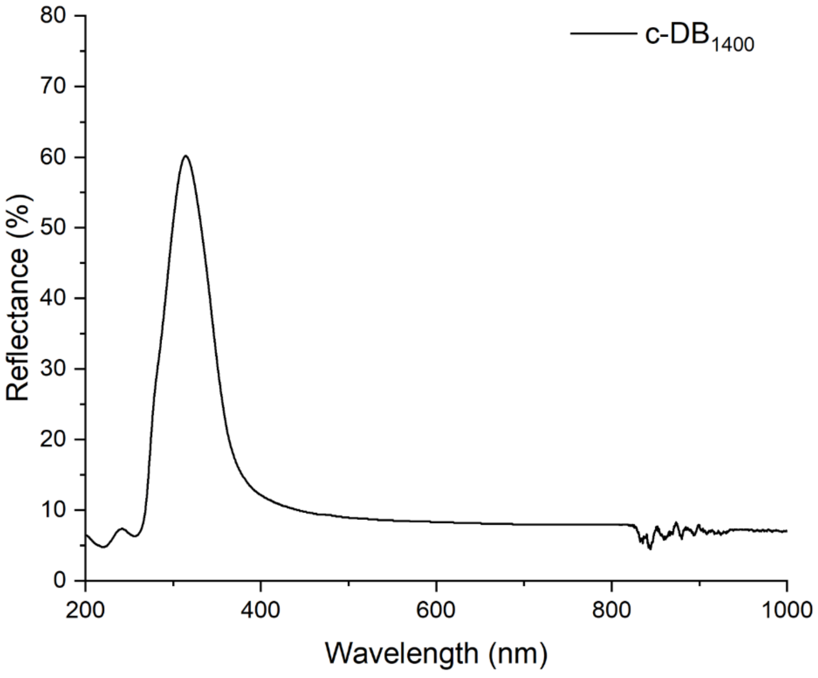

Figure S18. Plot of reflectance as a function of wavelength of c-DB_{1400}.

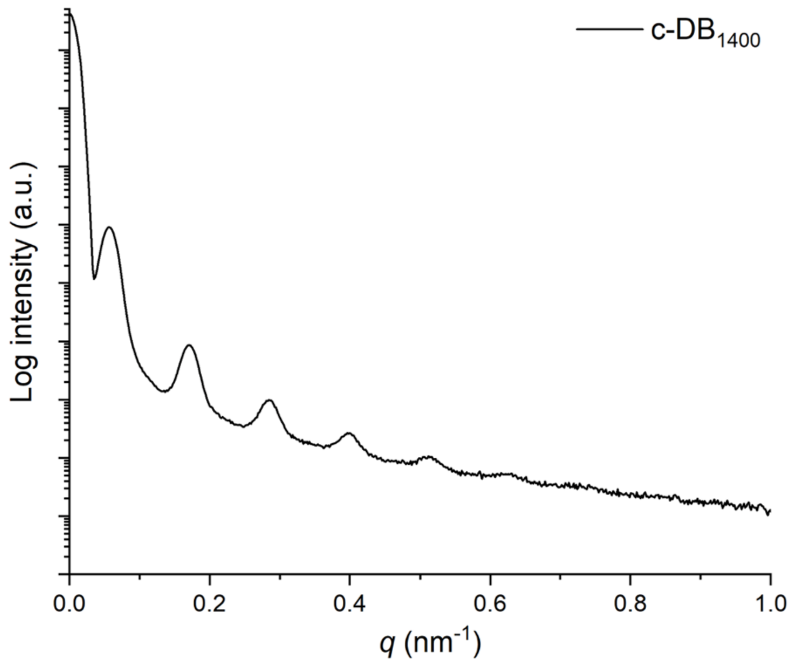

Figure S19. SAXS profile of c-DB_{1400}.

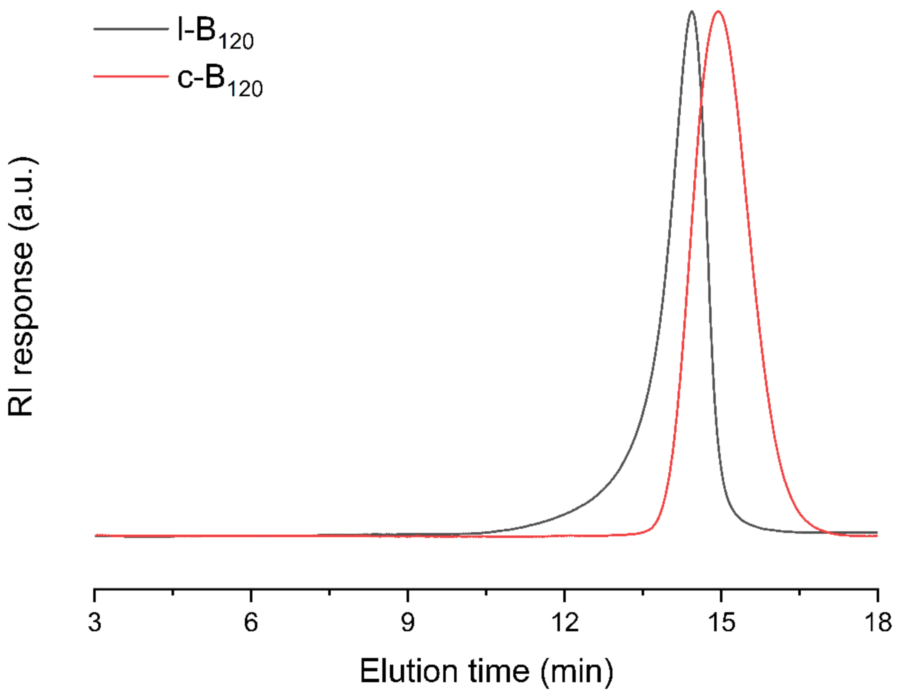

Figure S20. SEC traces of c-B_{120} and l-B_{120}.

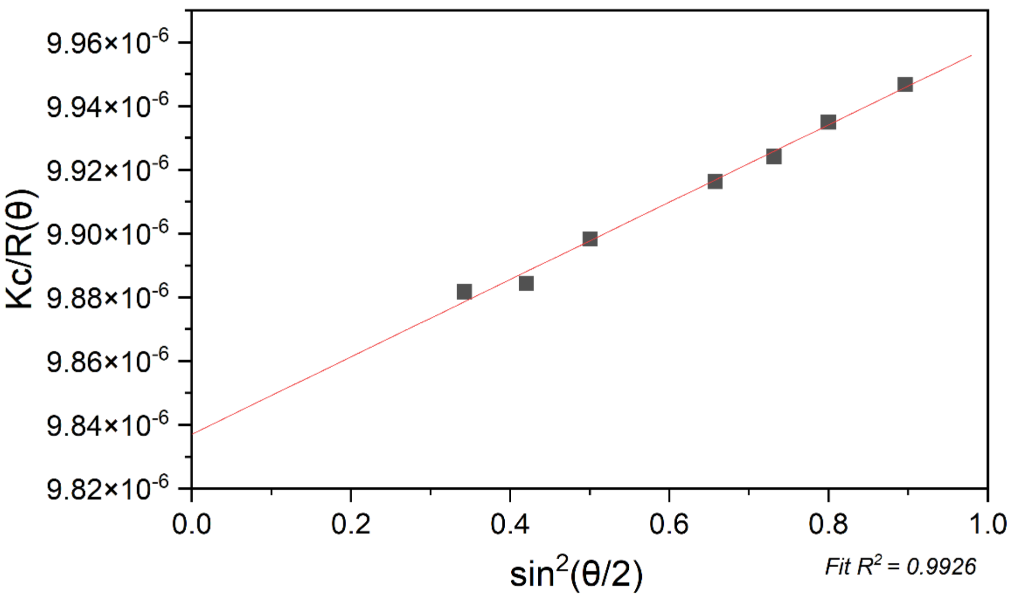

Figure 21. Kc/R(θ) plotted as a function of $\sin^2(\theta/2)$ for c-B_{120}, obtained from the angular dependence of the excess scattering for a single MALS-SEC elution slice at a polymer concentration of 2 mg mL^{-1} (estimated d*n*/d*c* from the elution slice = 0.1121 mL/g).

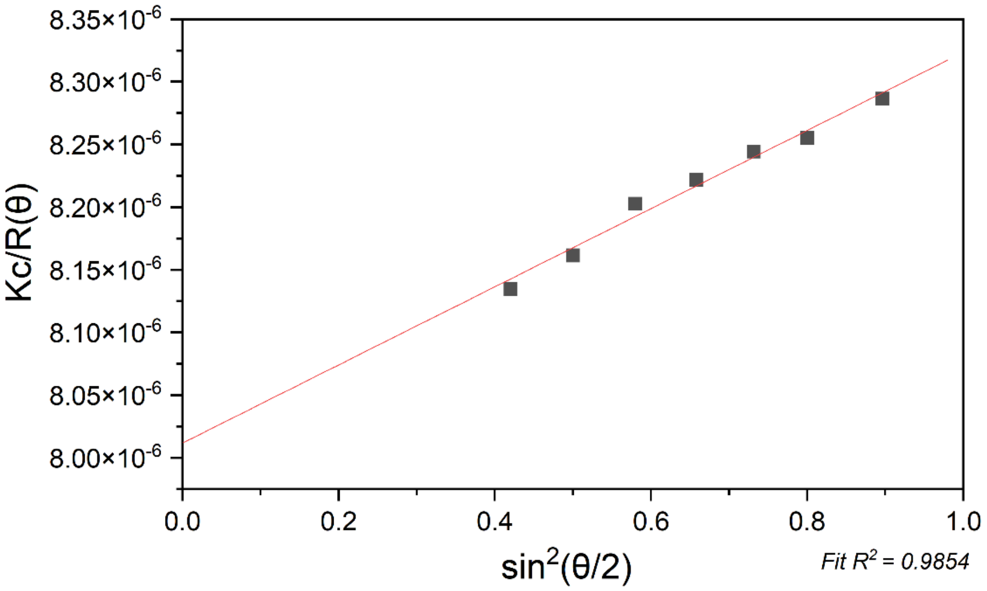

Figure S22. Kc/R(θ) plotted as a function of $\sin^2(\theta/2)$ for l-B_{120}, obtained from the angular dependence of the excess scattering for a single MALS-SEC elution slice at a polymer concentration of 2 mg mL^{-1} (estimated d*n*/d*c* from the elution slice = 0.1209 mL/g).

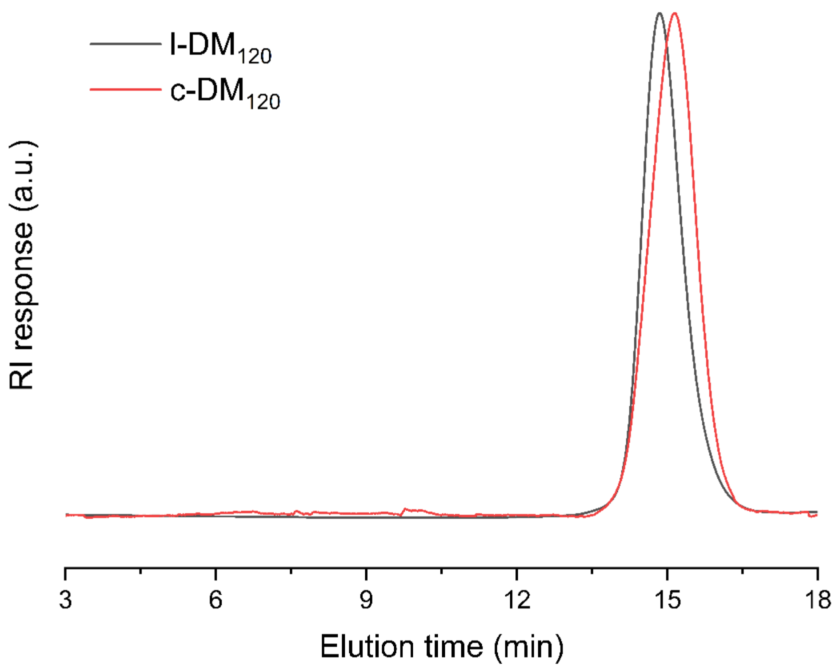

Figure S23. SEC traces of c-DM_{120} and l-DM_{120}.

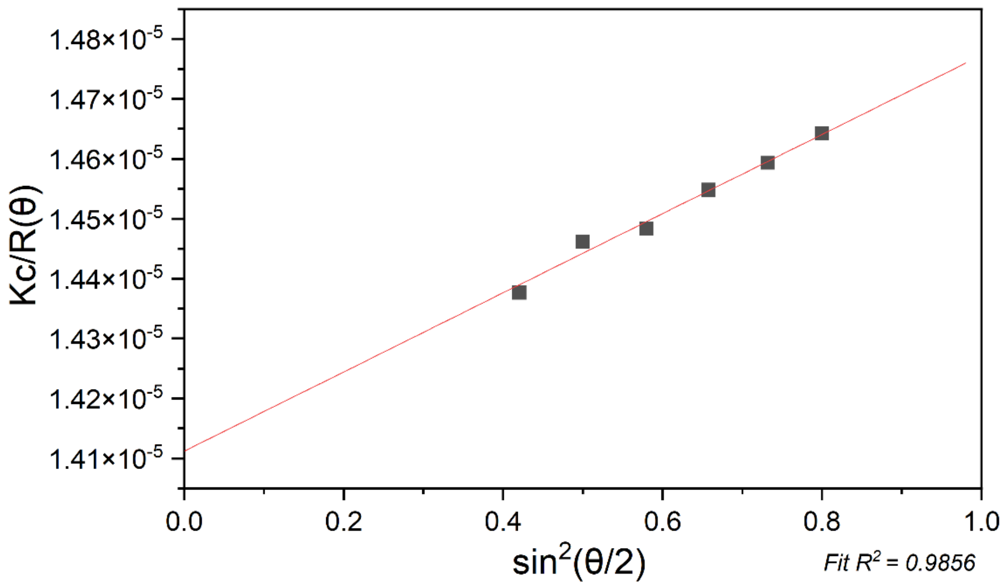

Figure S24. Kc/R(θ) plotted as a function of $\sin^2(\theta/2)$ for c-DM$_{120}$, obtained from the angular dependence of the excess scattering for a single MALS-SEC elution slice at a polymer concentration of 2 mg mL^{-1} (estimated d*n*/d*c* from the elution slice = 0.0260 mL/g).

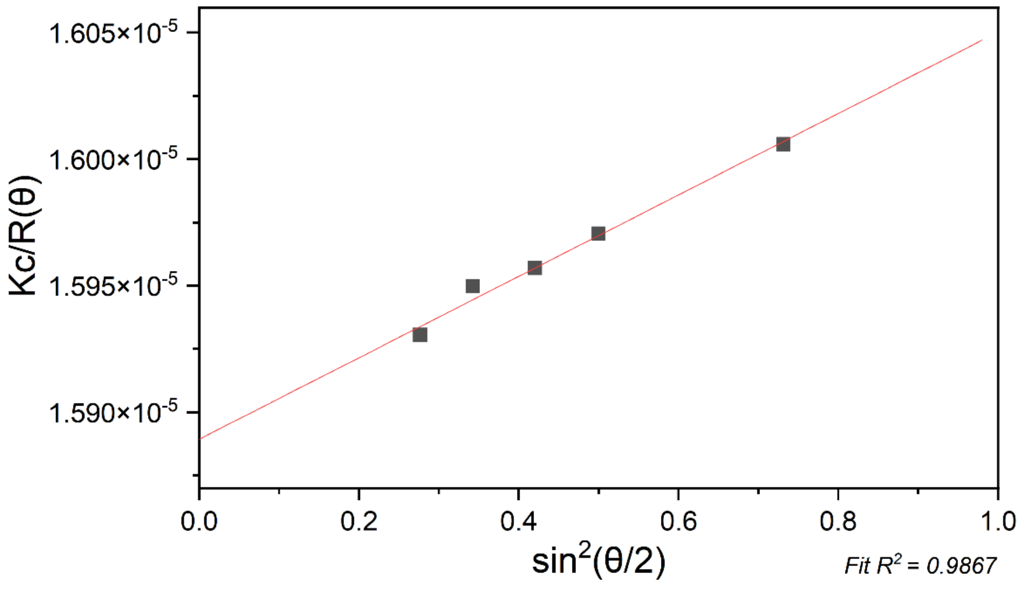

Figure S25. Kc/R(θ) plotted as a function of $\sin^2(\theta/2)$ for l-DM$_{120}$, obtained from the angular dependence of the excess scattering for a single MALS-SEC elution slice at a polymer concentration of 2 mg mL^{-1} (estimated d*n*/d*c* from the elution slice = 0.0372 mL/g).

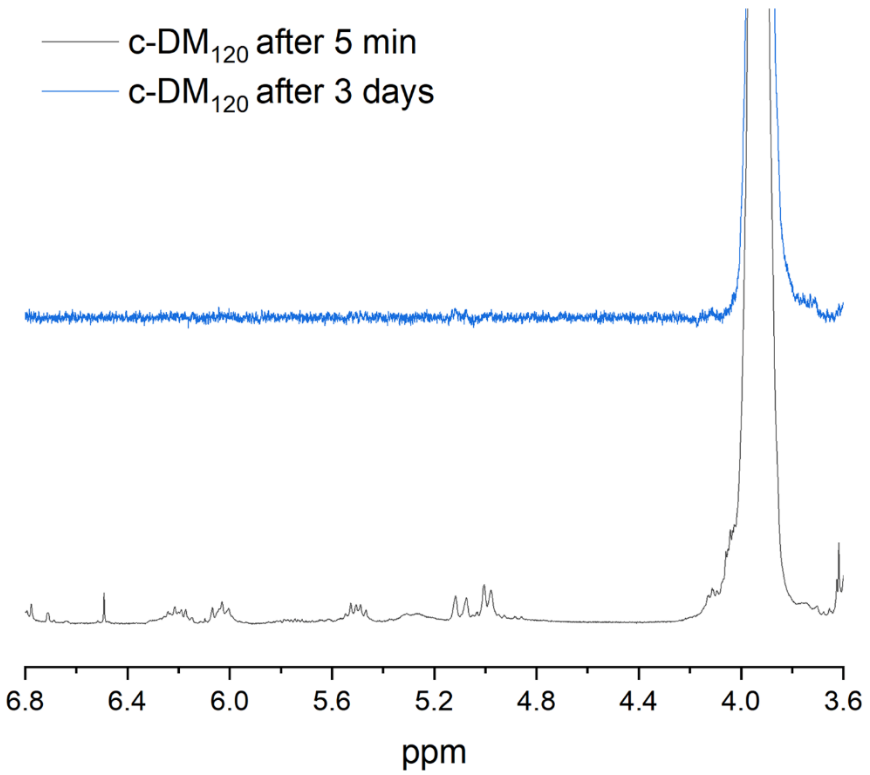

Figure S26. ^{1}H NMR spectral overlay ($CDCl_3$) of quenched c-DM_{120} after 5 min, after 3 days during LPP, showing that complete cyclization had occurred.

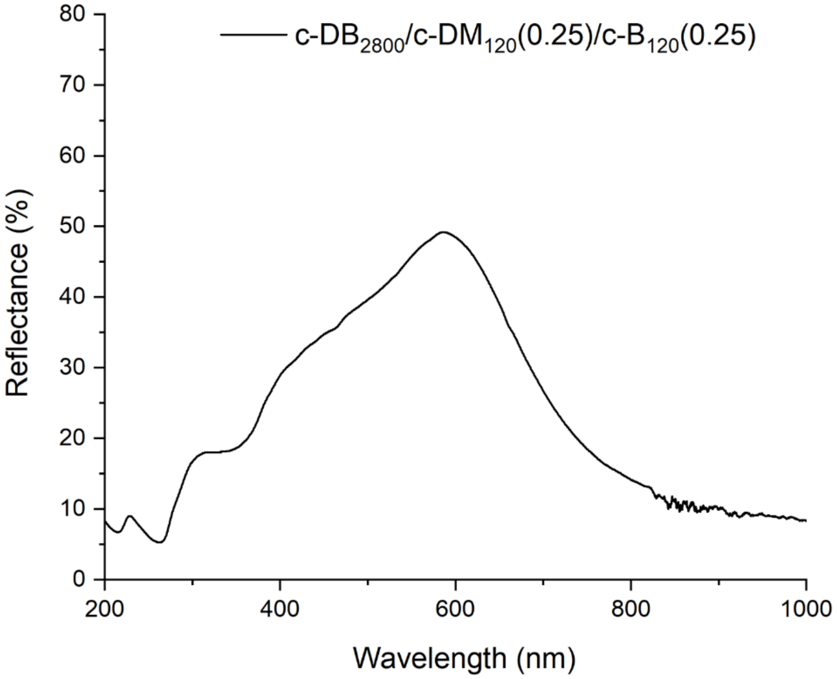

Figure S27. Plot of reflectance as a function of wavelength of c-DB_{2800}/c-DM_{120}(0.25)/c-B_{120}(0.25).

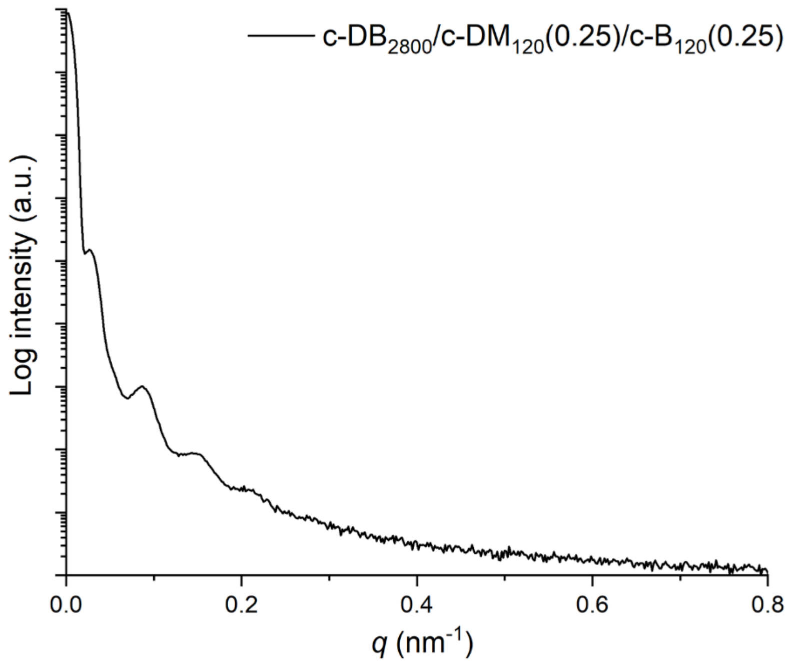

Figure S28. SAXS profile of c-DB_{2800}/c-DM_{120}(0.25)/c-B_{120}(0.25).

Table S1. Synthesis of CDCs, corresponding LDC counterparts, constituent CPs, and LPs via LPP

Entry	[DA]:[BnMA]:[MAD]:[LB]	Solvent	$[M]_0$ (M)	Conv. (%)
c-DB_{1400}	585:815:14:1	Toluene	0.3	> 99
l-DB_{1400}	585:815:14:1	Toluene	0.6	> 99
c-DB_{2800}	1017:1783:28:1	Toluene	0.6	> 99
l-DB_{2800}	1017:1783:28:1	Toluene	0.6	> 99
l-BDB_{2800}	1017:1783:28:1	Toluene	0.6	> 99
	[DA]:[MMA]:[MAD]:[LB]	Solvent	$[M]_0$ (M)	Conv. (%)
c-DM_{120}	84:36:2:1	Toluene	1.0	> 99
l-DM_{120}	84:36:2:1	Toluene	0.5	> 99
	[BnMA]:[MAD]:[LB]	Solvent	$[M]_0$ (M)	Conv. (%)
c-B_{120}	120:2:1	Toluene	0.5	> 99
l-B_{120}	120:2:1	Toluene	0.5	> 99
c-B_{40}	40:2:1	Toluene	0.5	> 99

Table S2. Characterization of CDCs, corresponding LDC counterparts, and constituent CPs

Entry	$M_{w,\ SEC}$ (kDa)^a	$Đ^a$	R_g (nm)	w_{PDA}	d (nm)
c-DB_{1400}	485.9	1.01	22.1	0.5	107
l-DB_{1400}	434.7	1.04	23.9	0.5	130
c-DB_{2800}	862.4	1.02	28.8	0.5	172
l-DB_{2800}	879.3	1.06	35.6	0.5	224
l-BDB_{2800}	832.3	1.07	35.7	0.5	180
c-DM_{120}	74.7	1.07	-	0.7	-
c-B_{120}	105.6	1.30	-	-	-
c-B_{40}	21.3	1.32	-	-	-
l-DM_{120}	65.8	1.07	-	0.7	-
l-B_{120}	121.9	1.03	-	-	-

^{a} Determined by SEC in $CHCl_3$ with a dn/dc method utilizing Wyatt DAWN HELEOS II 18-angle light scattering detector and a Wyatt Optilab TrEX dRI detector.

References

1. Shreve, A. P.; Mulhaupt, R.; Fultz, W.; Calabrese, J.; Robbins, W.; Ittel, S. D. Cobalt-Mediated Chain Transfer in the Controlled Polymerization of Methyl Methacrylate. *Organometallics* **1988**, *7* (2), 409–416.
